# Hominini-specific regulation of the cell cycle by stop codon readthrough of *FEM1B*

**DOI:** 10.1242/jcs.261921

**Published:** 2024-08-29

**Authors:** Md Noor Akhtar, Anumeha Singh, Lekha E. Manjunath, Dhruba Dey, Sangeetha Devi Kumar, Kirtana Vasu, Arpan Das, Sandeep M. Eswarappa

**Affiliations:** ^1^ Department of Biochemistry, Indian Institute of Science, Bengaluru 560012, India; ^2^ Undergraduate Program, Indian Institute of Science, Bengaluru 560012, India

**Keywords:** Cell cycle, FEM1B, stop codon, Translational readthrough

## Abstract

FEM1B is a substrate-recognition component of the CRL2 E3 ubiquitin-protein ligase. This multi-protein complex targets specific proteins for ubiquitylation, which leads to their degradation. Here, we demonstrate the regulation of *FEM1B* expression by stop codon readthrough (SCR). In this process, translating ribosomes readthrough the stop codon of *FEM1B* to generate a C-terminally extended isoform that is highly unstable. A total of 81 nucleotides in the proximal 3′UTR of *FEM1B* constitute the necessary and sufficient *cis*-signal for SCR. Also, they encode the amino acid sequence responsible for the degradation of the SCR product. CRISPR-edited cells lacking this region, and therefore SCR of *FEM1B*, showed increased FEM1B expression. This in turn resulted in reduced expression of SLBP (a target of FEM1B-mediated degradation) and replication-dependent histones (target of SLBP for mRNA stability), causing cell cycle delay. Evolutionary analysis revealed that this phenomenon is specific to the genus *Pan* and *Homo* (Hominini). Overall, we show a relatively recently evolved SCR process that relieves the cell cycle from the negative regulation by FEM1B.

## Teaser

**Summary:** Translation of *FEM1B* mRNA continues beyond its stop codon to generate a C-terminally extended unstable isoform, and this process regulates the cell cycle.

## INTRODUCTION

FEM1B is a component of the Cul2-RING (CRL2) E3 ubiquitin-protein ligase. This multi-protein complex, assembled on the cullin-2 scaffold, targets the degrons of specific proteins, and ubiquitylates them, resulting in their degradation (Koren et al., 2018; Wang et al., 2016). FEM1B provides substrate specificity in this process (Chen et al., 2021). CDK5R1, FNIP1, GLI1, ANKRD37, SMCR8 and SLBP are some of the proteins targeted by FEM1B-containing version of CRL2 (CRL2^FEM1B^) (Dankert et al., 2017; Gilder et al., 2013; Koren et al., 2018; Manford et al., 2020; Wang et al., 2016; Zhao et al., 2021). Through these targets, CRL2^FEM1B^ regulates multiple cellular functions including redox balance (via FNIP1) (Manford et al., 2020), oncogenicity (via GLI1) (Gilder et al., 2013) and the cell cycle (via SLBP) (Dankert et al., 2017). In addition to FEM1B, FEM1A and FEM1C can also function as substrate-recognition component of CRL2 (Chen et al., 2021).

Single nucleotide polymorphisms in *FEM1B* are associated with polycystic ovary syndrome (Goodarzi et al., 2008). Mice lacking *Fem1b* show impaired glucose tolerance due to defective glucose-mediated insulin secretion (Lu et al., 2005). FEM1B has a pro-apoptotic function in colon cancer cells, which is regulated by receptor for activated C kinase (RACK1) (Subauste et al., 2010, 2009). FEM1B has a CRL2-independent role in replication stress-induced checkpoint signalling (Sun and Shieh, 2009). In *Caenorhabditis elegans*, the homolog of FEM1B, Feminization-1 (FEM-1), promotes the poly-ubiquitylation and degradation of the sex-determining transformer protein 1 (TRA-1) to regulate the development of male phenotype (Starostina et al., 2007).

As CRL2 influences multiple cellular functions, it is imperative that its function is regulated. The constitutive photomorphogenesis 9 (COP9) signalosome complex inhibits the activity of CRL2 by removing NEDD8 from the activated cullin (Dubiel et al., 2020). Apart from this, the regulation of CRL2^FEM1B^ complex is poorly understood. In this study, we report regulation of *FEM1B* expression by stop codon readthrough (SCR) during translation. SCR is a process where ribosomes continue translation beyond a stop codon until the next available in-frame stop codon on the mRNA. Thus, a part of the 3′ untranslated region (UTR) gets translated during SCR resulting in a protein isoform with C-terminal extension (Manjunath et al., 2023). Basal SCR occurs at very low frequencies (<0.1%) in an mRNA due to translational errors. However, physiologically significant programmed SCR is observed at higher frequencies (>1%) in certain transcripts due to *cis*-acting RNA element downstream of the stop codon or *trans*-acting factors interacting with them (Firth et al., 2011; Jungreis et al., 2011). SCR has been observed in all domains of life (Bunnik et al., 2013; Jungreis et al., 2011; Sahoo et al., 2022; von der Haar and Tuite, 2007). This can change the function (e.g. *AGO1*), localization (e.g. *LDHB*) or stability (e.g. *MTCH2*) of the isoform depending on the nature of the C-terminus (Manjunath et al., 2020; Schueren et al., 2014; Singh et al., 2019; Stiebler et al., 2014). Previously, we reported that SCR of *MTCH2* regulates the level of MTCH2 protein by generating a highly unstable isoform that undergoes rapid proteosome-mediated degradation (Manjunath et al., 2020).

In this study, we demonstrate SCR in *FEM1B* mRNA, which results in an isoform that degrades quickly in a proteosome-dependent manner only in humans (genus *Homo*) and chimpanzees (genus *Pan*). We generated human cancer cell lines that lack SCR of *FEM1B* by genome editing. These cells exhibited reduced proliferation, clonogenicity and tumorigenicity. Our results show that this phenotype is because of the enhanced FEM1B expression caused by the absence of SCR in its mRNA. The increased expression of FEM1B led to reduced levels of stem loop binding protein (SLBP), which is a known target of FEM1B. This in turn resulted in reduced expression of replication-dependent histones, and cell cycle delay at S-phase. Thus, our study has unearthed an oncogenic translational event in *FEM1B* that regulates the cell cycle.

## RESULTS

### The proximal 3′UTR of *FEM1B* mRNA is important for normal cell cycle progression

**Fig. 1.**
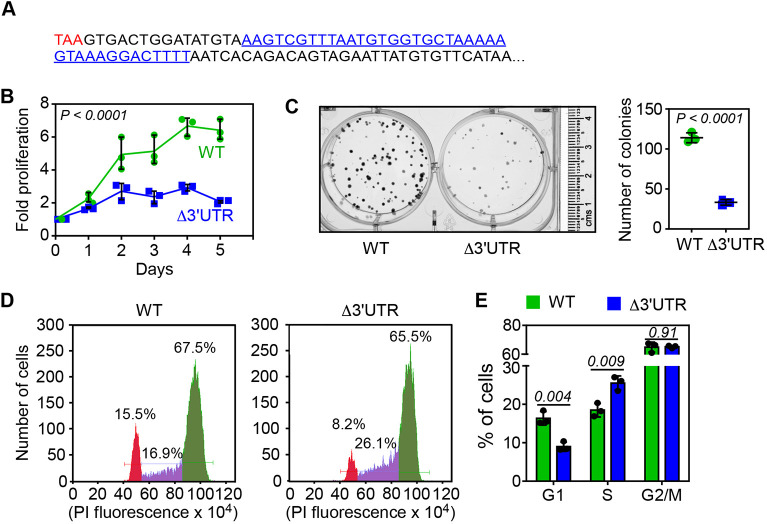
**The proximal 3**′**UTR of *FEM1B* mRNA is important for normal cell cycle progression**. (A) Sequence of the proximal 84 nucleotides of the 3′UTR of human *FEM1B*. The canonical stop codon is shown in red. The region shown in blue is deleted in Δ3′UTR HeLa cells. (B) Cell proliferation assay performed using MTT in wild-type (WT) and Δ3′UTR cells. Lines join the mean values of fold proliferation at 1st to 5th day relative to day 0 (*n*=3 biological replicates; error bars, s.d.). *P-*value, two-way ANOVA with no post test. (C) Representative images of clonogenicity assay (left). Quantification of colonies is shown in the graph (mean±s.d.; *n*=3 biological replicates). *P-*value, two-tailed unpaired Student's *t*-test. (D) Cell cycle analyses of cells 20 h after they were released from a double-thymidine block. Propidium Iodide-treated cells were analysed using a flow cytometer. The percentage (%) of cells belonging to G1 (red), S (purple), and G2/M (green) phases of cell cycle are shown. (E) The graph shows the quantification of the cell cycle analyses (mean±s.d.; *n*=3 biological replicates). Numbers inside the graph indicate *P-*values (two-tailed unpaired Student's *t*-test).

*FEM1B* mRNA has a long (4613 nucleotides in humans) 3′UTR. We observed that the proximal part of this 3′UTR is highly conserved across mammals from mouse to humans indicating its functional significance (Fig. S1). To investigate this, we deleted a 38-nucleotide stretch in this conserved region in HeLa cells (human cervical cancer cells) using the CRISPR-Cas9 system (Fig. 1A; Fig. S2A,B). These cells (termed Δ3′UTR) showed reduced proliferation compared to that in parental wild-type cells (Fig. 1B). A clonogenicity assay revealed that the ability of Δ3′UTR HeLa cells to form colonies was diminished (Fig. 1C). We analysed the cell cycle profile using the DNA-staining dye Propidium Iodide (Dankert et al., 2017). Cells were synchronized at the G1/S boundary by a double thymidine block (Chen and Deng, 2018). When released from this arrest, we observed a reduction in the percentage of Δ3′UTR cells under G1 phase with a concomitant increase in cells under S phase, compared to in the parental wild-type cells suggesting S-phase delay (Fig. 1D,E). To confirm these observations in another cell line, we deleted a portion of the conserved region in the proximal 3′UTR of *FEM1B* in MDA-MB-231 cells (human breast cancer cells) (Fig. S2C,D). These cells also showed reduced proliferation, reduced clonogenicity and cell cycle delay at the S-phase (Fig. S3A–D). Together, these results demonstrate the importance of the proximal 3′UTR in the execution of the cell cycle.

### The proximal 3′UTR of *FEM1B* mRNA regulates the expression of FEM1B

**Fig. 2.**
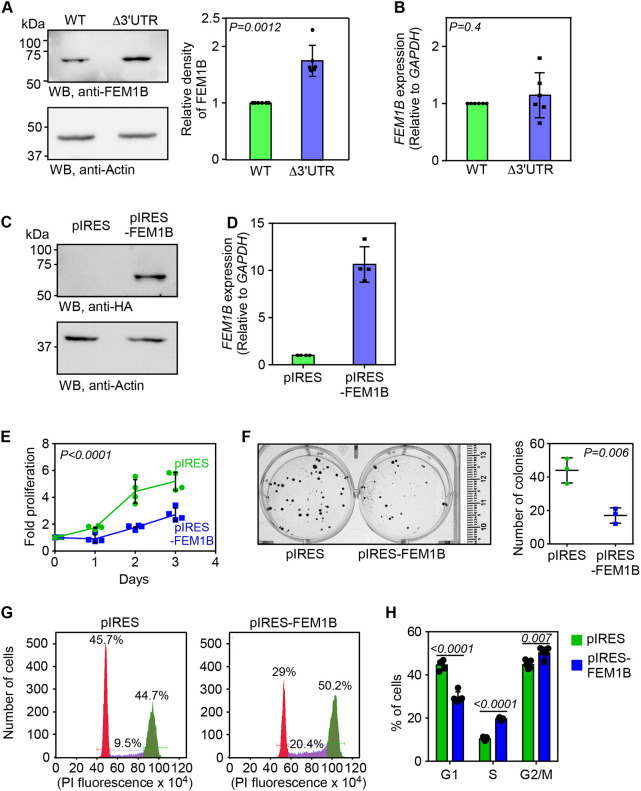
**The proximal 3**′**UTR of *FEM1B* mRNA determines the cellular levels of FEM1B, which regulates cell cycle.** (A) Western blot (WB) showing the expression of FEM1B in wild-type (WT) and Δ3′UTR HeLa cells. The graph shows the densitometry values (mean±s.d., *n*=6 biological replicates). (B) Expression of *FEM1B* relative to *GAPDH* calculated by qRT-PCR in wild-type and Δ3′UTR cells. Graph shows mean±s.d. (*n*=6 biological replicates). *P-*values in A and B were calculated by two-tailed paired *t*-test. (C) Western blot showing the expression of exogenous HA-tagged FEM1B in cells stably expressing pIRES-FEM1B. Blot is representative of three repeats. (D) Expression of *FEM1B* relative to *GAPDH* calculated by qRT-PCR. Graph shows mean±s.d. (*n*=4 technical replicates). (E) Cell proliferation assay performed using MTT. Lines join the mean values of fold proliferation at 1st to 3rd day relative to day 0 (*n*=4 biological replicates; error bars, s.d.). *P-*value, two-way ANOVA with no post test. (F) Representative images of clonogenicity assay (left). Quantification of colonies is shown in the graph (mean±s.d.; *n*=3 biological replicates). *P-*value, two-tailed unpaired Student's *t*-test. (G) Cell cycle analyses of cells 20 h after they were released from double thymidine block. Propidium iodide-treated cells were analysed using a flow cytometer. The percentage (%) of cells belonging to G1 (red), S (purple) and G2/M (green) phases of cell cycle are shown. (H) The graph shows the quantification of the cell cycle analyses (mean±s.d.; *n*=4 biological replicates). Numbers inside the graph indicate *P-*values (two-tailed unpaired Student's *t*-test).

3′UTRs are known to regulate the expression of their mRNAs by influencing their stability or translation (Mayr, 2019). We tested the expression of FEM1B in Δ3′UTR HeLa cells described above. We observed a significant increase in the expression of FEM1B in these cells compared to that in parental wild-type cells (Fig. 2A). However, there was no significant change in the *FEM1B* mRNA levels in these cells (Fig. 2B). These findings suggest that the proximal 3′UTR of *FEM1B* regulates its expression at a post-transcriptional level. To confirm these observations, we used Δ3′UTR MDA-MB-231 cells, which also showed enhanced FEM1B expression at protein level, but not at the mRNA level (Fig. S3E–G).

### Enhanced FEM1B expression causes cell cycle delay

We investigated whether enhanced FEM1B expression was responsible for the cell cycle delay observed in Δ3′UTR cells. For this, we generated HeLa cells stably overexpressing *FEM1B* cloned into the pIRES vector, which was confirmed by western blotting and quantitative real-time PCR (qRT-PCR) analyses (Fig. 2C,D). Like Δ3′UTR cells (Fig. 1), FEM1B-overexpressing cells showed reduced proliferation, reduced clonogenicity and S-phase delay in the cell cycle (Fig. 2E–H). These observations show that the cell cycle is regulated by the levels of FEM1B, which is in turn are regulated by the proximal 3′UTR of its mRNA.

**Fig. 3.**
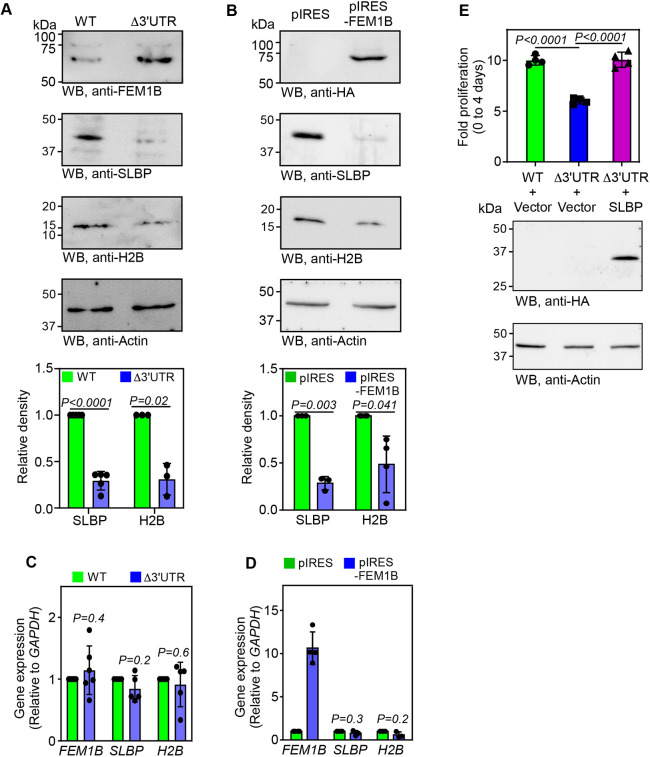
**Cellular levels of FEM1B regulates SLBP, and H2B expression.** (A) Western blot (WB) showing the cellular levels of FEM1B, SLBP and H2B in wild-type (WT) and Δ3′UTR HeLa cells. The graph below shows the densitometry values [mean±s.d., *n*=5 (SLBP) or 3 (H2B) biological replicates]. (B) Western blot showing the cellular levels of FEM1B, SLBP and H2B in HeLa cells stably overexpressing HA-tagged FEM1B. The graph below shows the densitometry values [mean±s.d., *n*=3 (SLBP) or 4 (H2B) biological replicates]. (C,D) Expression of *FEM1B*, *SLBP* and *H2B* relative to *GAPDH* calculated by quantitative real-time PCR in wild-type and Δ3′UTR cells (C), and cells overexpressing HA-tagged FEM1B (D). *FEM1B* expression data shown in C and D are the same as those shown in Fig. 2B and D, respectively. (E) Cell proliferation assay performed using MTT in wild-type and Δ3′UTR cells transiently overexpressing HA-tagged SLBP or empty vector (pCDNA3.1B). The graph shows fold proliferation at day 4. Graphs represent mean±s.d. *n*=4 biological replicates, except for *FEM1B* expression in D, where technical replicates are shown. *P-*values were calculated by two-tailed paired *t*-test (A–D), or two-tailed unpaired Student's *t*-test (E).

Stem loop binding protein (SLBP) is a target of FEM1B-mediated degradation (Dankert et al., 2017). SLBP binds conserved stem loops in the 3′UTR of replication-dependent histone mRNAs that lack the poly-A tail. Through this interaction, SLBP positively regulates the expression of replication-dependent histone mRNAs at post-transcriptional level. Thus, SLBP is essential for normal cell cycle progression (Sullivan et al., 2009; Zhao et al., 2004). We explored this pathway in Δ3′UTR cells. We observed a decrease in the levels of SLBP and histone proteins H2B and H4 in Δ3′UTR cells compared to what was seen in parental wild-type cells (Fig. 3A; Fig. S3E,F, S4A,B). H2B and H4 are replication-dependent histone proteins whose expression is regulated by SLBP (Sullivan et al., 2009; Zhao et al., 2004). Similarly, HeLa cells overexpressing FEM1B also showed reduced expression of SLBP, H2B and H4 proteins compared to control cells (Fig. 3B; Fig. S4D,E). There was no significant change in the levels of *SLBP*, *H2BC8* (encoding H2B) and *H4C2* (encoding H4) mRNAs in any of these cells indicating post-transcriptional regulation (Fig. 3C,D; Fig. S3G, S4C,F). Overexpression of SLBP in Δ3′UTR cells increased their proliferation comparable to wild-type cells (Fig. 3E). Together, these results show that the cell cycle delay observed in Δ3′UTR cells was because of increased FEM1B expression leading to reduced levels of SLBP, H2B and H4 proteins required for normal S-phase progression.

### The proximal 3′UTR of *FEM1B* mRNA is translated by SCR

**Fig. 4.**
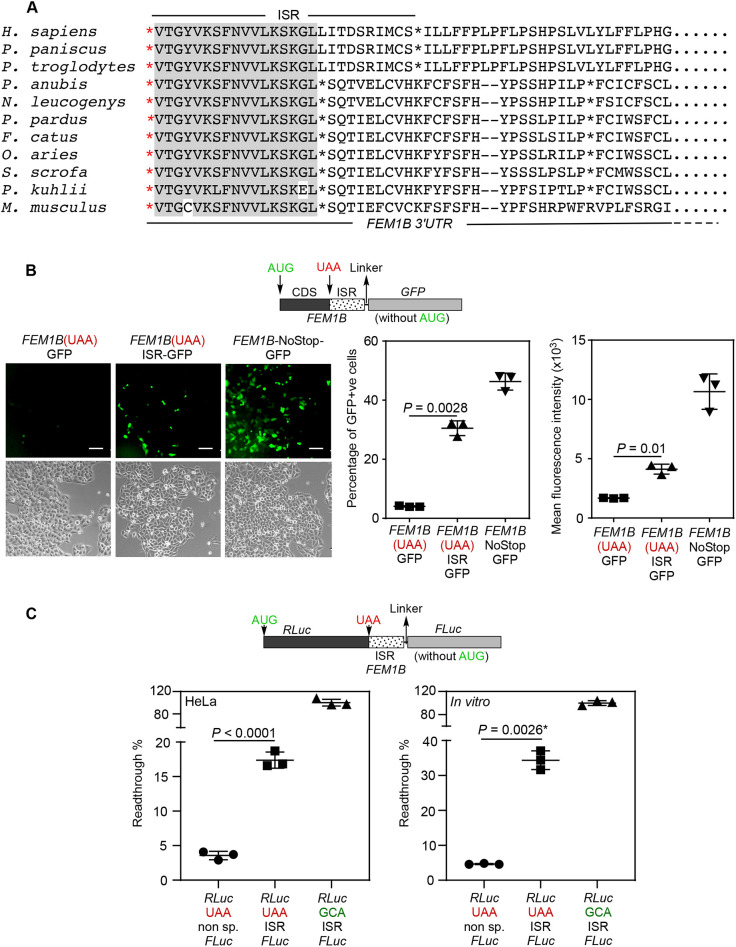
***FEM1B* mRNA exhibits stop codon readthrough (SCR).** (A) Alignment of amino acid sequences predicted to be encoded by the proximal 3′UTR of *FEM1B* from 11 mammalian species. Amino acids conserved across all species are shown with a grey background. In-frame stop codons are indicated by asterisks. ISR, inter-stop codon region. (B) Fluorescence-based SCR assay. Schematic shows the plasmid construct used in the assay (CDS, coding sequence of *FEM1B*; GFP, green fluorescent protein). Fluorescence microscopy images show the expression of GFP in HeLa cells transfected with the indicated constructs. Scale bars: 100 µm. The graphs show quantification of fluorescence in these cells by flow cytometry (mean±s.d.; *n*=3 biological replicates). (C) Luminescence-based SCR assay. Schematic shows the construct used in the assay. RLuc, *Renilla* luciferase; FLuc, firefly luciferase. Non sp., non-specific sequence. Graphs show the results of SCR assay performed in HeLa cells and *in vitro* using rabbit reticulocyte lysate. SCR activity is indicated as a percentage of SCR occurring. The relative luciferase activity of GCA construct was taken as 100% (mean±s.d.; *n*=3 biological replicates). *P-*values in all graphs were calculated by two-tailed unpaired Student's *t*-test.

Our next goal was to understand the mechanism of regulation of *FEM1B* expression by its proximal 3′UTR. We observed that the amino acid sequence potentially encoded by the proximal 3′UTR of *FEM1B* mRNA is highly conserved across mammals. Also, there is a downstream stop codon in frame with the canonical stop codon. The location of the downstream stop codon is also partially conserved across mammals (Fig. 4A). These observations indicate that the proximal 3′UTR might get translated by SCR (Eswarappa et al., 2014; Jungreis et al., 2011).

To test this hypothesis, we employed a fluorescence-based SCR assay (Manjunath et al., 2020). The cDNA for GFP was cloned downstream of the partial coding sequence (CDS) of human *FEM1B* along with its stop codon and the first 81 nucleotides of its 3′UTR. This 81-nucleotides stretch is termed the inter-stop codon region (ISR) as it lies between the canonical stop codon and the downstream in-frame stop codon (Fig. S1; Fig. 4A,B). The start codon of the *GFP* was removed such that GFP was expressed only if there was SCR across the canonical stop codon of *FEM1B*. We observed a significant fluorescence in HeLa cells transfected with this construct, much above the background signal, indicating that SCR occurred. The fluorescence was quantified using a flow cytometer. The cells transfected with the test construct [*FEM1B*(UAA)ISR-*GFP*] showed a higher percentage of GFP-positive cells and higher values of mean fluorescence intensity compared to the cells transfected with a construct without the 3′UTR [*FEM1B*(UAA)-*GFP*], which provided the background fluorescence signal. Another construct without any stop codon between *FEM1B* and *GFP* [*FEM1B*-NoStop-*GFP*] was used as a positive control, which showed a strong fluorescence as expected (Fig. 4B).

Next, we employed a luminescence-based SCR assay (Singh et al., 2019). Here, *GFP* cDNA in the plasmid construct described above was replaced by the cDNA of firefly luciferase (*FLuc*) (Fig. S5A). The activity of firefly luciferase (i.e. luminescence) was expected from these constructs only if there was SCR across the stop codon of *FEM1B*. We performed *in vitro* transcription followed by *in vitro* translation using rabbit reticulocyte lysate. As for the fluorescence-based assay, and consistent with there being *FEM1B* SCR, we observed significant luminescence from this construct, which was much above the background level (Fig. S5B). Similar observations were made in HeLa cells transfected with these constructs. A plasmid expressing *Renilla* luciferase (RLuc) was used as transfection control and the SCR was measured by determining the FLuc activity relative to the RLuc activity (Fig. S5C; Table S4). We then mutated the canonical stop codon UAA to other two stop codons in this construct. The UGA stop codon showed more SCR compared to other two stop codons (Fig. S5D; Table S5). This is expected as UGA is the leakiest stop codon. Together, these fluorescence- and luminescence-based assays demonstrate SCR of *FEM1B* mRNA.

We analysed the publicly available mass spectrometry data to detect peptides corresponding to the ISR of *FEM1B*, which will provide strong evidence for SCR of endogenous *FEM1B*. Analysis using MaxQuant identified the peptide GLLITDSR in primary human testicular peritubular cells with a score of 143.37 (Fig. S6A) (PXD033534; Stepanov et al., 2022). Notably, testes show the highest *FEM1B* transcript expression among all human tissues (Human Protein Atlas; https://www.proteinatlas.org/). The peptide GLLITDSR is unique to the SCR product of *FEM1B,* and no other part of the human genome encodes this peptide.

Ribosome profiling reveals the regions of an mRNA that are translated. Ribosomal footprints on the proximal part of the 3′UTR provide evidence for SCR, and this has been used to identify novel SCR events (Dunn et al., 2013; Sahoo et al., 2022). We analysed ribosome profiling data available in RiboCrypt (https://ribocrypt.org/), an interactive visualization online tool. We employed this tool to visualize ribosome footprints on *FEM1B* mRNA (Transcript ID: ENST00000306917) from all ribosome profiling samples available there (all_merged-Homo_sapiens). Consistent with SCR, we observed ribosome footprints beyond the canonical stop codon until second in-frame stop codon (Fig. S6B). Similar observations were made in samples derived from human testis and HeLa cells (Fig. S6C,D). These observations also provide evidence for SCR of endogenous *FEM1B*.

### The ISR of *FEM1B* is necessary and sufficient to induce SCR

To test if the ISR (the first 81 nucleotides of the 3′UTR) of *FEM1B* alone can drive the SCR in a heterologous system, we employed a dual luciferase-based SCR assay (Grentzmann et al., 1998). The ISR of *FEM1B* was cloned between the cDNAs of *RLuc* and *FLuc.* In this construct, the expression of *RLuc* was mediated by canonical cap-mediated initiation of translation, and the expression of *FLuc* was possible only if there was SCR across the stop codon of *RLuc* driven by the ISR of human *FEM1B* (schematic in Fig. 4C). The ratio of FLuc activity to RLuc activity (relative luciferase activity) provided us with the efficiency of SCR. HeLa cells transfected with this construct showed a significantly higher relative luciferase activity compared to that in cells transfected with control construct (a nonspecific sequence in place of the ISR). Similar observations were made by *in vitro* translation of mRNAs obtained by *in vitro* transcription of these constructs. Readthrough efficiency was higher in *in vitro* conditions compared to that in cells, suggesting that this process is regulated inside cells (Fig. 4C; Table S1). These results show that the ISR of human *FEM1B* is responsible for SCR. A construct without any stop codon between RLuc and Fluc was used to obtain the maximum relative luciferase activity and to calculate the efficiency of SCR. In HeLa cells, the *FEM1B* ISR was able to induce SCR across the stop codon of RLuc with an efficiency of 17%, and *in vitro* the same was 34%, which are both much above the basal levels caused by translation errors (Fig. 4C; Table S2).

### The SCR of *FEM1B* generates a highly unstable isoform called FEM1Bx

**Fig. 5.**
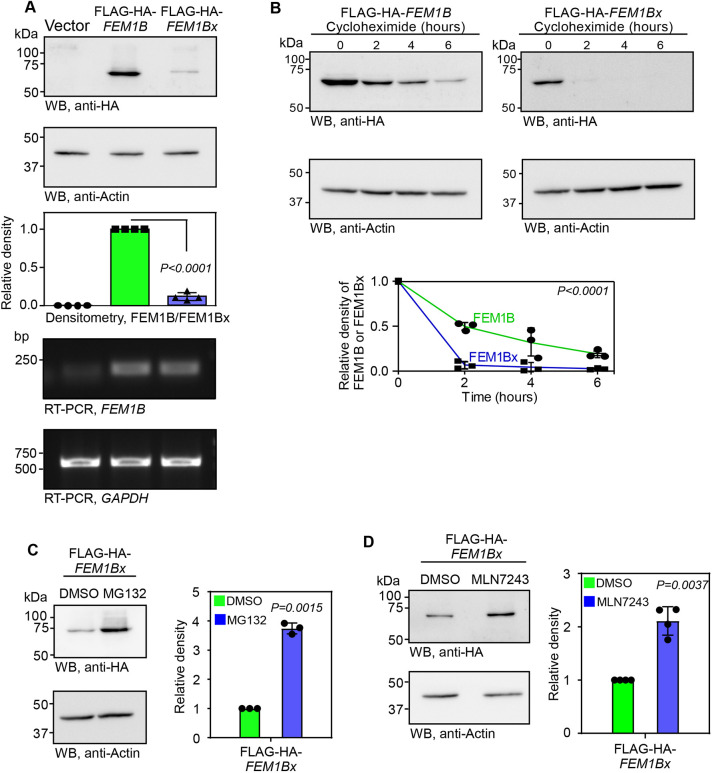
**The SCR of *FEM1B* generates a highly unstable isoform.** (A) Results of western blotting (WB) and RT-PCR analyses showing the expression of FLAG–HA–FEM1B and FLAG–HA–FEM1Bx at protein and mRNA levels in HeLa cells. Assays were performed 24 h after transfection. (B) Western blots showing the levels of FLAG–HA–FEM1B and FLAG–HA–FEM1Bx in HeLa cells treated with cycloheximide (100 µg/ml) for 2, 4 and 6 h. (C,D) Western blot showing the expression of FLAG–HA–FEM1Bx in HeLa cells treated with MG132 (10 µM for 10 h) (C) or MLN7243 (5 µM for 6 h) (D). The graphs show the mean±s.d. density of the bands (*N*=3 in B and C; 4 in A and D). *P*-values, two-way ANOVA with no post test (B) or two-tailed paired *t*-test (A,C,D).

After demonstrating SCR, we investigated the function of the SCR product, which we named FEM1Bx (x for extended isoform). We tried to exogenously overexpress FEM1Bx in HeLa cells to test its effect on cell cycle. In this construct, the canonical stop codon of *FEM1B*, UAA, was mutated to CAA to maximize the expression of FEM1Bx. To our surprise, we were unable to achieve robust expression of FEM1Bx, unlike FEM1B, which showed very good expression. However, the expression of *FEM1B* and *FEM1Bx* were comparable at mRNA level (Fig. 5A). We then investigated the stability of these two protein isoforms by treating cells with cycloheximide. Whereas FEM1B was observed until 6 h after the treatment, FEM1Bx was undetectable just 2 h after the treatment (Fig. 5B). These results show that FEM1Bx is an unstable isoform. Treatment of cells with MG132, a proteosome inhibitor, was able to rescue the stability of FEM1Bx (Fig. 5C). Enhanced expression of FEM1Bx was also observed in cells treated with MLN7243, an inhibitor of the ubiquitin-activating enzyme (Hyer et al., 2018) (Fig. 5D). These results indicate the involvement of ubiquitin-dependent proteosome system in the degradation of FEM1Bx.

We generated an antibody that recognizes the peptide (VTGYVKSFNVVLKSKGL) encoded by the proximal part of the ISR of *FEM1B,* termed the anti-FEM1Bx antibody. The anti-FEM1Bx antibody was able to detect the synthetic peptide VTGYVKSFNVVLKSKGL, but not a control peptide. Importantly, the antibody could not detect the peptide when it was pre-incubated with the peptide (Fig. S7A). Furthermore, the anti-FEM1Bx antibody was able to detect the overexpressed exogenous SCR product FEM1Bx in HEK293 cells, which also demonstrates SCR (Fig. S7B). After confirming the specificity of the antibody, we then used it to detect the endogenous FEM1Bx. FEM1Bx was undetectable in many cell lines, including HeLa cells, which is in agreement with the poor stability of this isoform. However, anti-FEM1Bx antibody was able to detect a band between 75 and 63 kDa in HepG2 cells (Fig. S7C). To confirm that this band is derived from *FEM1B*, we electroporated HepG2 cells with *FEM1B*-targeting shRNAs. These shRNAs reduced the expression of the canonical isoform FEM1B and the band detected by anti-FEM1Bx antibody. This observation shows that the band detected by the antibody represents FEM1Bx (Fig. S7D). Similarly, this antibody was able to detect FEM1Bx in MDA-MB-231 cells after a prolonged exposure, which was absent in Δ3′UTR cells (Fig. S7E). Furthermore, the peptide GLLITDSR, specific to the ISR of *FEM1B*, was detected in samples derived from endogenous HepG2 cells (Fig. S7F) (PXD029268; Ben-Haim et al., 2021). These observations show evidence for the endogenous FEM1Bx and suggest that its stability is regulated in a cell type-specific manner.

### Increase in endogenous SCR of *FEM1B* decreases its expression and activity

**Fig. 6.**
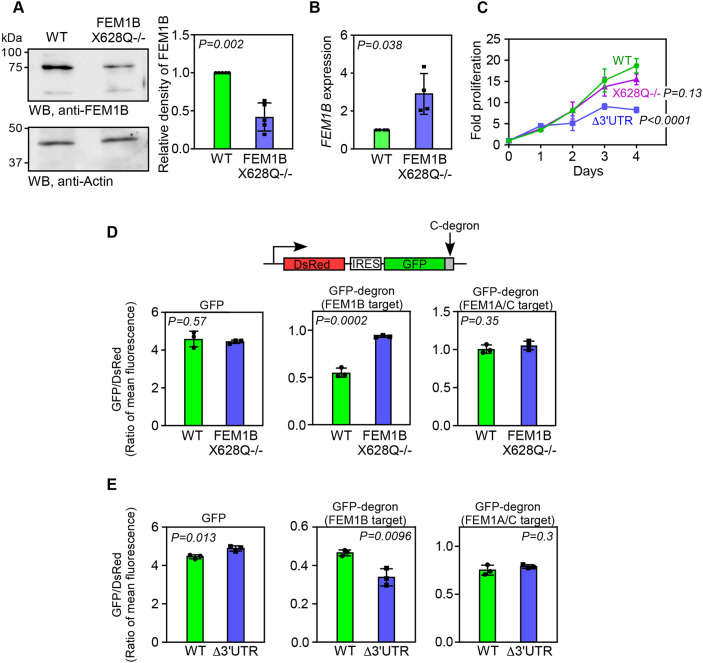
**Mutation of the canonical stop codon of *FEM1B* reduces FEM1B level.** (A) Expression of FEM1B in wild-type (WT) and FEM1B^X628Q−/−^ cells, where the canonical stop codon of *FEM1B* UAA was mutated to CAA. The graph shows the results of the densitometry analysis (mean±s.d., *n*=5 biological replicates). (B) Expression of *FEM1B* relative to *GAPDH* calculated by qRT-PCR in wild-type and FEM1B^X628Q−/−^ cells. The graph shows mean±s.d. (*n*=4 biological replicates). *P-*values were calculated using a two-tailed paired *t*-test. (C) Cell proliferation assay performed using MTT. Lines join the mean values of fold proliferation at 1st to 4th day relative to day 0 (*n*=3 biological replicates; error bars, s.d.). *P-*values, two-way ANOVA with no post test. (D,E) Results of dual fluorescence-based protein stability assay performed in wild-type and FEM1B^X628Q−/−^ cells (D), or Δ3′UTR cells (E). Schematic of the constructs used is shown. The stability of GFP with or without C-terminal degron is denoted by the ratio of mean fluorescence intensity of GFP to that of DsRed. Graphs show mean±s.d. (*n*=3 biological replicates). *P-*values were calculated using two-tailed unpaired Student's *t*-test.

To test the stability of endogenous FEM1Bx, we generated HeLa cells where the canonical stop codon of *FEM1B,* UAA, was mutated to CAA by genome editing using the CRISPR-Cas9 system (Fig. S2E). These cells (FEM1B^X628Q−/−^) generate FEM1Bx constitutively as the canonical stop codon of *FEM1B* was mutated. Western blotting revealed an ∼50% decrease in protein levels in these mutant cells compared to their parental wild-type cells (Fig. 6A). This observation is consistent with the SCR of endogenous *FEM1B* and the poor stability of its product. We also observed an increase in *FEM1B* mRNA levels in FEM1B^X628Q−/−^ cells, which could be due to a possible feedback regulation at transcriptional level (Fig. 6B). Unlike Δ3′UTR cells, FEM1B^X628Q−/−^ cells did not show reduced cell proliferation suggesting that reduced levels of (or even the absence of) the canonical isoform of *FEM1B* do not affect the cell cycle (Fig. 6C).

We then investigated the FEM1B activity in FEM1B^X628Q−/−^cells where levels of FEM1B isoforms were reduced by ∼50% (Fig. 6A). For this, we employed a fluorescence-based assay as described previously (Chen et al., 2021; Yen et al., 2008). This assay utilizes a construct expressing two fluorescent proteins from the same mRNA. First, *Discosoma* sp. red fluorescent protein (DsRed) is expressed by canonical translation initiation, and it serves as an internal control. After that, enhanced GFP with the degron to be tested is expressed via an internal ribosome entry site (IRES) (schematic in Fig. 6D). We appended the sequence YKKRLLLGLDR at the C-terminus of the GFP; this sequence is a C-terminal degron and known target of CRL2^FEM1B^ (Chen et al., 2021). As a control, we appended the sequence YSVNSLLKELR at the same location; this sequence is a C-terminal degron and known target of CRL2^FEM1A^ and CRL2^FEM1C^ (CRL2^FEM1A/C^) (Chen et al., 2021). These constructs were transfected in HeLa cells and the relative GFP fluorescence (ratio of the mean fluorescence intensity of GFP to that of DsRed) was measured from DsRed-positive cells by flow cytometry. This serves as an indicator of the stability of GFP. We observed an increase in the relative fluorescence of GFP tagged with CRL2^FEM1B^ degron (YKKRLLLGLDR) in FEM1B^X628Q−/−^ cells, where FEM1B levels were low. And there was a decrease in the relative fluorescence of GFP tagged with CRL2^FEM1B^ degron in Δ3′UTR cells, which showed higher FEM1B levels (Fig. 6D,E). There was no change in the relative fluorescence in these cells when the GFP was tagged with CRL2^FEM1A/C^ degron (YSVNSLLKELR). These results are consistent with the regulation of FEM1B expression by SCR.

### The peptide sequence generated by *FEM1B* ISR is sufficient to induce protein degradation

We tested whether the 27 amino acid sequence encoded in the *FEM1B* ISR was enough to cause the degradation of a protein. We cloned *FEM1B* ISR downstream of *GFP* cDNA such that GFP is generated with the C-terminal extension (27 amino acids) of FEM1Bx. When transfected in HeLa cells, GFP with C-terminal extension was expressed at very low level compared to GFP without any extension. This observation was made by fluorescence microscopy. Another plasmid construct where a stop codon was inserted between the sequences of *GFP* and *FEM1B* ISR exhibited robust GFP expression, showing that translation of the *FEM1B* ISR is required for the protein instability (Fig. S8A,B). We performed a protein stability assay using cycloheximide, which blocks translation. We observed robust expression of GFP without any extension even after 3 h of cycloheximide treatment. However, GFP with the FEM1Bx extension showed a substantially reduced expression just after 1 h of treatment. Insertion of a stop codon between the coding sequence of GFP and the ISR was enough to bring back the stability of GFP (Fig. S8C). Furthermore, MG132 was able to rescue the instability of GFP–ISR showing the involvement of proteasomal complex in its degradation (Fig. S8D). These results show that the peptide encoded by *FEM1B* ISR is sufficient to cause proteasomal degradation of the protein to which it is appended.

### Generation of an unstable isoform by the SCR of *FEM1B* mRNA is a recently evolved phenomenon

**Fig. 7.**
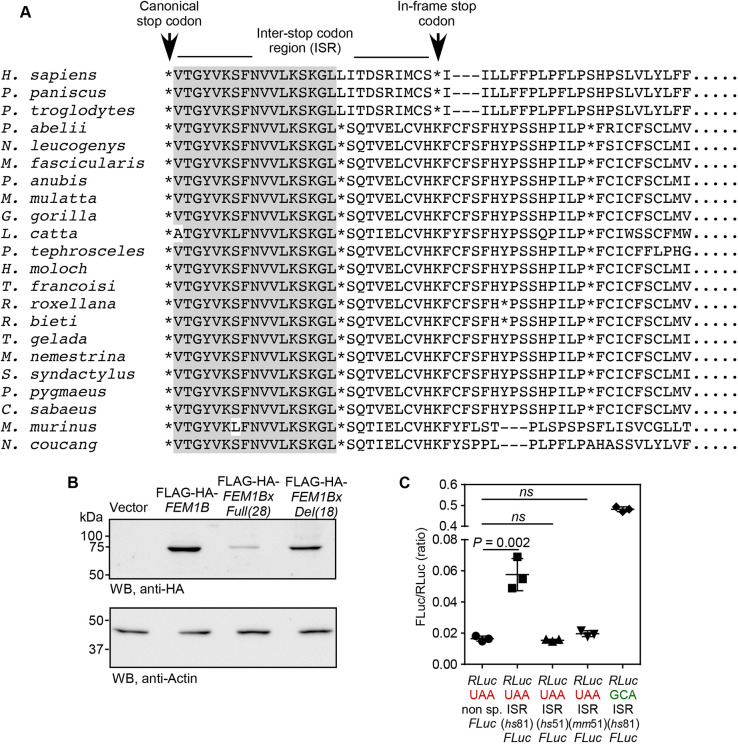
**Generation of an unstable isoform by the SCR of *FEM1B* mRNA is a recently evolved phenomenon.** (A) Alignment of amino acid sequences potentially encoded by the proximal 3′UTR of *FEM1B* from 22 primates. Amino acids conserved across all species are shown with a grey background. In-frame stop codons are indicated by asterisks. All primate *FEM1B* ISR sequences currently available in NCBI are included in this analysis. (B) Expression of FLAG–HA-tagged human full-length FEM1Bx (28 amino acids extension) and truncated FEM1Bx (18 amino acids extension) corresponding to non-hominid ISR sequence in HeLa cells. Blot is representative of three repeats. (C) Results of luminescence-based SCR assay (schematic in Fig. 4C) performed in HeLa cells using constructs with full-length human ISR (hs81), the proximal 51 nucleotides of human ISR (hs51) and mouse ISR (mm51). SCR activity is indicated as firefly luciferase activity relative to *Reniila* luciferase activity (mean±s.d. of FLuc/RLuc; *n*=3 biological replicates). *P-*value, two-tailed unpaired Student's *t*-test. ns, not significant.

FEM1Bx is 28 amino acids (27 from the ISR and 1 from the stop codon) longer than FEM1B because of the C-terminal extension generated by SCR in humans (genus *Homo*) and chimpanzees (genus *Pan*), the only extant members of the tribe Hominini under the order primates. However, in other mammals, including primates such as gorillas, gibbons and orangutans, SCR of *FEM1B*, if it happens, could generate only 18 amino acids extension (Figs 4A and 7A). This is because of the insertion of a ‘T’ at the 53rd nucleotide position after the canonical stop codon in humans and chimpanzees, which changes the translation frame (Fig. S1). The shorter version of FEM1Bx, corresponding to the ISR of other mammals (18 amino acids) was more stable than the human FEM1Bx (Fig. 7B). Furthermore, unlike human *FEM1B* ISR (81 nts), the mouse *Fem1b* ISR (51 nts) and the corresponding truncated human *FEM1B* ISR (51 nts) were unable to induce SCR in dual luciferase-based assays (Fig. 7C; Table S3). These observations suggest that the regulation of *FEM1B* expression by SCR has evolved relatively recently when the evolutionary path of the last common ancestor of humans and chimpanzees was separated from the path leading to gorillas.

### Functional significance of the regulation of FEM1B expression by SCR

**Fig. 8.**
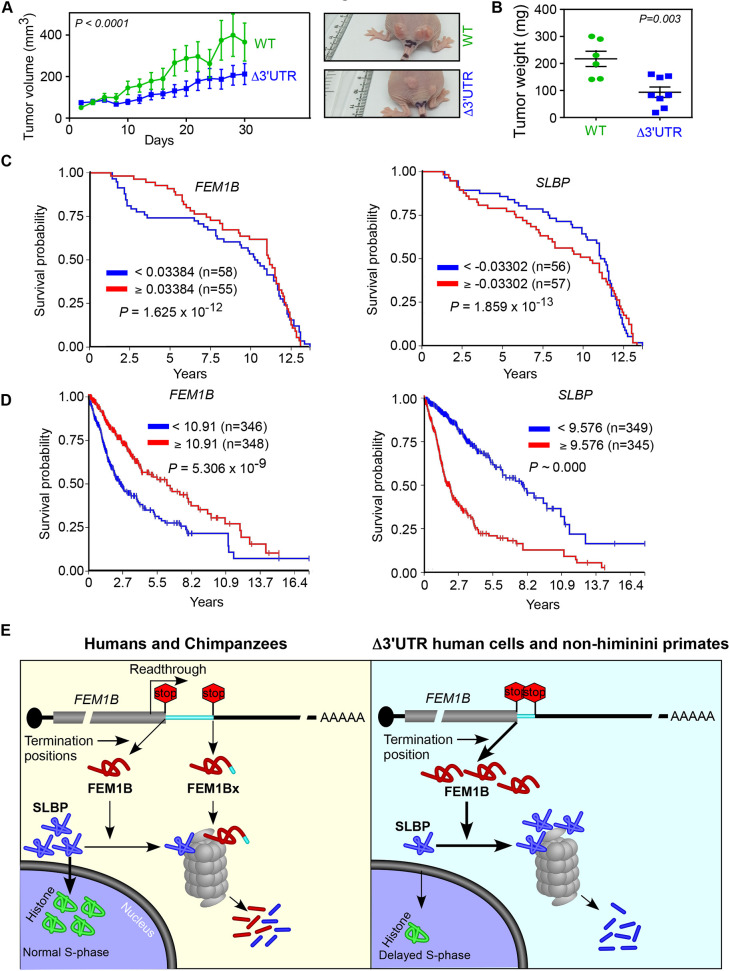
**The levels of FEM1B regulates cancer progression.** (A) Growth of xenograft tumours derived from HeLa cells [wild-type (WT) and Δ3′UTR] in athymic nude mice. The tumour progression was monitored using digital Vernier callipers by calculating the tumour volume [(shortest diameter)^2^×(longest diameter)×0.5] (mean±s.e.m.; *n*=10 tumours). *P-*value, two-way ANOVA test with no post test. Representative images of the tumours are shown. (B) The graph shows the weight of tumours after 30 days [mean±s.e.m. *n*=6 (for WT) and 8 (for Δ3′UTR) tumours]. *P-*value, two-tailed unpaired Student's *t*-test. (C,D) Kaplan–Meier curves showing the correlation between the probability of survival of cancer patients (breast cancer in C, and low-grade glioma and glioblastoma in D and the expression of *FEM1B* or *SLBP*. The analyses were performed using the Xena Functional Genomics Explorer. Red and blue lines indicate high and low expression of *FEM1B* or *SLBP*. The default cut off values set by the explorer that defined high or low expression are shown. (E) Regulation of cell cycle by SCR of *FEM1B* mRNA. *FEM1B* mRNA has two conserved in-frame stop codons. Translation termination at the first stop codon generates the canonical isoform, FEM1B, which is a substrate recognition component of the CRL2 E3 ubiquitin-protein ligase. One of the targets of CRL2^FEM1B^ is SLBP, which positively regulates the expression of replication-dependent histone proteins. This pathway ensures normal progression of S-phase of the cell cycle. *FEM1B* mRNA exhibits SCR in humans and chimpanzees resulting in an unstable isoform termed FEM1Bx. Therefore, the SCR keeps a check on the levels of FEM1B. In the absence of SCR, as in non-Hominini primates, the FEM1B level is high, which causes reduced levels of SLBP due to increased degradation mediated by CRL2^FEM1B^. This in turn causes decrease in the levels of replication-dependent histone proteins required for the normal progression of S-phase of the cell cycle resulting in cell cycle delay.

We investigated the physiological significance of SCR of *FEM1B* using xenograft tumour model in athymic nude mice. We subcutaneously inoculated Δ3′UTR HeLa cells, and their wild-type parental cells in the flanks of athymic nude mice. It should be noted that Δ3′UTR cells (HeLa and MDA-MB-231) showed enhanced expression of FEM1B (Fig. 2A; Fig. S3E). Inoculated cells formed visible tumours in a week. We monitored the progression of the tumours by measuring their volume. In agreement with the cell cycle phenotype, xenograft tumours formed by Δ3′UTR cells were slower growing compared to those formed by wild-type cells (Fig. 8A). After 30 days, we euthanized the mice and dissected the tumours. Tumours formed by Δ3′UTR cells had less mass than those formed by wild-type cells (Fig. 8B).

We then analysed the correlation between survival of human cancer patients with the expression of FEM1B using Xena Functional Genomics Explorer (https://xenabrowser.net/). We generated Kaplan–Meier survival curves, which plots the probability of survival of patients with high and low expression of a given gene, which was *FEM1B* in our case. Consistent with the results of cell cycle analysis and the tumour xenograft experiments, there was a significant increase in the probability of survival in breast cancer patients with higher expression of *FEM1B*. Interestingly, there was an opposite correlation with the expression of *SLBP* in the same dataset. Patients with lower expression of *SLBP* showed better chances of survival (Fig. 8C). Similar observations were made for lower grade glioma and glioblastoma patients from The Cancer Genome Atlas (TCGA) (Fig. 8D). Together, these observations demonstrate a role of FEM1B levels in cancer progression.

## DISCUSSION

The 3′UTR of an mRNA plays an important role in regulating its expression. It can serve as a target for stabilizing or destabilising RNA-binding proteins. In mammalian cells, microRNAs primarily target 3′UTRs resulting in mRNA degradation and/or translation inhibition. Sequences in the 3′UTRs of some mRNAs fold into *cis*-acting secondary structures, such as stem loops and pseudoknots, which can regulate translation or stability. In addition to these, translation in the 3′UTR by SCR or by internal initiation in downstream open reading frames (dORFs) in the 3′UTR, can bring about regulatory effects on the expression of the mRNA (Mayr, 2017, 2019).

Our results suggest that the proximal 3′UTR of *FEM1B* regulates its expression. SCR of *FEM1B* translates its proximal 3′UTR, generating a longer unstable isoform with a C-terminal extension, termed FEM1Bx, which is prone to proteasomal degradation. Ironically, FEM1B, which recognizes the C-terminus of its target proteins for degradation, gets easily degraded by its own C-terminal extension generated by SCR.

The stability of FEM1Bx is improved upon treatment with MG132 (a proteosome inhibitor) or MLN7243 (an inhibitor of the ubiquitin-activating enzyme) treatment, suggesting the involvement of ubiquitin-dependent proteasome system in the degradation of FEM1Bx (Fig. 5C,D). In the absence of SCR, as observed in Δ3′UTR cells, the cellular levels of the canonical FEM1B increases, as this is the only isoform that can be generated from the *FEM1B* mRNA in these cells (Fig. 8E). This will in turn generate more CRL2^FEM1B^ protein-degrading complexes, which will degrade its target SLBP more efficiently. Reduced SLBP level causes reduced expression of replication-dependent histones, resulting in cell cycle delay. It is possible that the role of FEM1B in the replication stress-induced checkpoint signalling also plays a role in the cell cycle delay observed in Δ3′UTR cells (Sun and Shieh, 2009).

Thus, it appears that SCR of *FEM1B* serves as a mechanism that has evolved to partially relieve the cell cycle from the clutches of FEM1B. Remarkably, the SCR-mediated regulation of FEM1B expression appears to be present only in humans (genus *Homo*) and chimpanzees (genus *Pan*). This is because of a single nucleotide insertion in the proximal 3′UTR of *FEM1B* in these primates, which might have occurred ∼10 million years ago when the branch leading to humans and chimpanzees was separated from the one that lead to gorillas (Figs S1 and S7) (Scally et al., 2012). This could partly explain why humans are more susceptible to cancer than other primates (Huang et al., 2022; Puente et al., 2006). In support of this, we observed a positive correlation between FEM1B expression and survival in certain cancer patients. Furthermore, there was a negative correlation between SLBP expression and survival in same cohort of patients. Although these analyses are based on mRNA expression, they indicate the clinical significance of FEM1B and SLBP protein levels in tumour progression, which can be regulated by SCR. In agreement with this, cells with higher FEM1B expression showed reduced tumorigenicity (Fig. 8A). Furthermore, a stop-to-sense variant in *FEM1B* (Mutation ID, COSM1729087; c.1883A>T; p.*628L; pathogenicity score, 0.9796), which would rapidly degrade the protein, has been observed in hepatocellular carcinoma (http://NonStopDB.dkfz.de). Therefore, we propose that FEM1B and SLBP levels can potentially be used as markers to predict the prognosis of certain cancers. Interestingly, a stop-to-sense variant in the tumour suppressor gene *SMAD4*, leading to its rapid degradation, has been observed in multiple cancers (Dhamija et al., 2020).

The process of SCR is driven by *cis*-factors in the 3′UTR immediately downstream of the stop codon, such as stem loops or pseudoknots (Firth et al., 2011; Jungreis et al., 2011). In some cases, SCR is positively regulated by *trans*-factors binding to the 3′UTR immediately downstream of the stop codon, such as microRNAs and proteins (Eswarappa et al., 2014; Singh et al., 2019). The SCR of *FEM1B* is driven by the 81-nucleotide-long ISR. This region can drive the SCR in a heterologous context as also shown by our dual-luciferase assay. Although the efficiency of SCR based on reporter assays in HeLa cells was determined as ∼17%, this is likely to be an underestimate as the SCR product is highly unstable (Fig. 5). The actual SCR efficiency of endogenous *FEM1B* is likely to be higher in these cells, as complete absence of SCR resulted in nearly doubling of FEM1B levels (Δ3′UTR cells, Fig. 2A and Fig. S3E), and constitutive SCR resulted in nearly halving of FEM1Bx levels (FEM1B^X628Q−/−^ cells, Fig. 6A). We also observed cell type-specific expression of FEM1Bx. The expression was high in HepG2 cells, low in MDA-MB-231 cells and undetectable in other cell types tested (Fig. S7). This suggests that there is tissue-specific regulation of SCR process and/or the regulation of the stability of FEM1Bx. Further investigations are needed to identify the factors responsible for the *FEM1B* SCR process and its regulation.

## MATERIALS AND METHODS

### Cell culture

HeLa, MDA-MB-231, HEK293 and HepG2 cells (Indian Institute of Science, India except for HepG2, which were from the National Centre for Cell Sciences, India) were cultured in Dulbecco's modified Eagle's medium (DMEM, HiMedia) containing 10% fetal bovine serum (FBS, Gibco) and 1% antibiotics (10,000 units/ml penicillin, 10,000 µg/ml streptomycin, Lonza) (complete medium) at 37°C in a humidified atmosphere containing 5% CO_2_. Identity of the cells was confirmed by STR profiling. Cells were tested for mycoplasma contamination twice a year.

### Antibodies and chemical reagents

Antibodies used were as follows: anti-FEM1B (1:1000 Sigma, AV53702 or HPA041920); anti-HA (1:3000; Sigma, 11867423001); anti-GFP (1:5000; BioLegend, 902602); anti-SLBP (1:1000; Abcam, ab181972); anti-H2B (1:1000; Santa Cruz Biotechnology, sc-515808); anti-H4 (1:1000; Santa Cruz Biotechnology, sc-25260); and anti-actin (1:30,000; Sigma, A3854). Horseradish peroxidase-conjugated secondary antibodies were from Thermo Scientific. All antibodies were used at concentrations recommended by the manufacturer. MLN7243 was from Cayman Chemicals (30108). MG132 was from Abcam (ab141003). Thymidine (T1895), cycloheximide (01810) and Thiazolyl Blue Tetrazolium Bromide, MTT (M5655) were all from Sigma. We are happy to share all the reagents used in this study.

### RT-PCR

Total RNA was isolated from cells using RNAiso Plus (TaKaRa) as per the manufacturer's instructions. The concentration and the quality of the RNA were measured with a BioPhotometer (Eppendorf). cDNA synthesis was performed using 0.5–1 µg RNA and RevertAid Reverse Transcriptase (Thermo Fisher Scientific) as per the manufacturer's instructions. For semi-quantitative RT-PCR, amplification was undertaken for 28 cycles using gene-specific primers. Quantitative real-time PCR was carried out using SYBR Green mix (TaKaRa) and a CFX Opus 96 instrument (Bio-Rad laboratories). PCR conditions were as follows: initial denaturation at 95°C for 5 min; followed by 40 cycles of denaturation at 95°C for 30 s, annealing at 55°C for 30 s and extension at 72°C for 30 s; and a final extension at 72°C for 5 min. This was followed by melt curve analysis. Quantification was performed by the 2^−ΔΔCt^ method.

Sequences of primers (5′ to 3′) used for RT-PCR were: *GAPDH* (for semi-qRT-PCR), CCACCCATGGCAAATTCCATGGCA and TCTAGACGGCAGGTCAGGTCAGGTCCACC; *GAPDH* (for semi- and qRT-PCR), ACAACTTTGGTATCGTGGAAGG and GCCATCACGCCACAGTTTC; *H2BC8*, ACAAGCGCTCGACCATTACCT and TGGTGACAGCCTTGGTACCTTC; *SLBP*, CGCAGACCCGAGAGCTTTA and TGCCATTTCTTTGTTAACTCTGGT; *EGFP*, AAGTTCATCTGCACCACCG and TCCTTGAAGAAGATGGTGCG; *FLUC*, CAACTGCATAAGGCTATGAAGAGA and ATTTGTATTCAGCCCATATCGTTT; *FEM1B*, AGTCTCAAGTGCCTGGCTGC and TGAACACATAATTCTACTGTC; and *H4C2*, GGATAACATCCAAGGCATCACC and CGCCACGAGTCTCCTCATAAAT.

### Deletion of the proximal part of the 3′UTR of *FEM1B*

The sgRNAs (5′-ATATGTAAAGTCGTTTAATG-3′ and 5′-CATGGTAATTGATTTCAGAC-3′) targeting the proximal part of the 3′ untranslated region (UTR) of *FEM1B* gene were cloned into the pSpCas9(BB)-2A-GFP (PX458) plasmid (Addgene #48138). The sgRNAs were selected based on their high target score and low off-target score compared to others (https://www.benchling.com/crispr). A total of 1 µg per well of each sgRNA-expressing plasmid, which also expresses Cas9, was transfected in a 24-well plate using Lipofectamine 2000. GFP-positive cells were sorted and seeded at a density of one cell per well in a 96-well plate after 48 h of transfection using FACSAria^TM^ II sorter (BD Biosciences). The clones were expanded and screened for the deletion by PCR using a pair of primers flanking the target region of *FEM1B* (5′-CCGCTAGACAAAAGTACAACTGG-3′ and 5′-TGTCCCTTGTTACATAAACAAAACA-3′). The deletion was confirmed by PCR of genomic DNA and sequencing of the PCR product.

### Colony formation assay

Cells harvested from an exponentially growing culture were seeded in a six-well plate at a very low density (∼100 cells per well). Cells were allowed to grow and expand into colonies. After colonies were formed, they were gently washed with PBS and fixed using 6% glutaraldehyde. The colonies were stained using 0.5% Crystal Violet (Sigma, C0775). After 30 min, the stain was removed, and the plate was rinsed in water. Plates were then dried and imaged for counting the number of colonies.

### Cell proliferation assay

Cells were seeded (∼ 5000 cells per well) in a 96-well plate. At different time points (0 to 5 days), MTT [3-(4,5-dimethylthiazol-2-yl)-2,5-diphenyl tetrazolium bromide] reagent (50 µg/well) was added to the cells in the medium. After 2 h, the medium was replaced by 100 µl of DMSO and incubated for 15 min. The supernatant was then transferred to another 96-well plate, and the absorbance at 575 nm was measured using a microplate reader (VERSAmax, Molecular Devices). The fold proliferation was calculated by taking the ratio of the absorbance at a given time point to that on day zero.

### Cell cycle analysis

Cells were seeded at 20–30% confluence (∼ 50,000 cells per well) in a six-well plate and incubated overnight. Cells were then treated with 2 mM thymidine for 16 h. Cells were washed three times with PBS and incubated with fresh medium without thymidine for 9 h. Cells were again treated with 2 mM thymidine for another 16 h. This double-thymidine treatment arrests the cells at G1/S boundary in the cell cycle (Chen and Deng, 2018). For cell cycle analysis, arrested cells were released by treating them with complete medium. After 20 h (HeLa cells) or 30 h (MDA-MB-231 cells), cells were harvested and pelleted down by centrifugation at 700 ***g***. Cells were fixed using ice-cold 70% ethanol after a wash and incubated at −20°C overnight. Fixed cells were treated with RNase A (20 µg/ml; Thermo Fisher Scientific, EN0531) at 37°C for 1 h after removing the fixative by centrifugation and washing multiple times with PBS. Cells were washed again and treated with Propidium Iodide (10 µg/ml; Invitrogen, P3566) at room temperature for 30 min. They were subjected to cell cycle analysis by flow cytometry (CytoFLEX S, Beckman Coulter). The data analysis to calculate the percentage of cells in different phases was performed using the CytExpert software (Beckman Coulter).

### Western blotting

Cells lysis was undertaken using cell lysis buffer (20 mM Tris-HCl pH 7.4, 150 mM NaCl, 1 mM EDTA and 1% Triton X-100) with protease inhibitor (Promega). Electrophoresis of cell lysates was carried out in a 10%, 12% or 15% SDS-polyacrylamide gel for 1.5 to 2 h. Proteins were then transferred onto a PVDF membrane (Immobilion-P; Merck Millipore) using Trans-Blot semi-dry transfer apparatus (Bio-Rad) at constant current. The blot was blocked using 5% skimmed milk for 1 h at room temperature and then incubated with the primary antibody overnight, followed by the horseradish peroxidase-conjugated secondary antibody. The blot was then developed using Clarity ECL reagent (Bio-Rad) or Femto ECL reagent (Thermo Scientific or Giri Diagnostics), and the images were recorded using LAS-3000 imager (Fujifilm) or BioRad Chemdoc^TM^. The band densities were quantified using the ImageJ software. Background-deducted density of a test protein band was normalized to that of actin (loading control) to calculate the relative density. Equal areas of the bands were taken for quantification. Uncropped western blot images are provided in Fig. S9.

### SCR assays

The partial coding sequence (CDS) of *FEM1B* (534 nucleotides of the 3′ end) with its canonical stop codon and the inter-stop codon region (ISR) was cloned upstream of the CDS of firefly luciferase (FLuc) or green fluorescent protein (GFP) in the pcDNA3.1B vector (Invitrogen). The downstream in-frame stop codon of *FEM1B* and the start codon of FLuc/GFP were excluded from the construct such that FLuc/GFP was expressed only if there was translational readthrough across the canonical stop codon of *FEM1B*. A linker sequence (GGCGGCTCCGGCGGCTCCCTCGTGCTCGGG) was inserted between *FEM1B* and FLuc/GFP. Another construct without the ISR was used as a control to measure the fluorescence due to basal SCR resulting in the background luminescence/fluorescence. A construct without any stop codon between *FEM1B* and *FLuc/GFP* was used to calculate the efficiency of SCR.

Lipofectamine 2000 was used for transfection in all experiments. For luminescence-based assays, transfected HeLa cells were lysed 24 h after transfection using 1× passive lysis buffer (Promega). Luminescence was measured in a Glomax Explorer (Promega) using the Dual-Luciferase Reporter Assay System (Promega). For *in vitro* translation assays, the FLuc constructs were linearized and *in vitro* transcribed using T7 RNA polymerase. Obtained RNA was purified and an equal amount was used for *in vitro* translation using the Rabbit Reticulocyte Lysate system (Promega). Luciferase activity was calculated as explained above.

For fluorescence-based SCR assay, GFP reporter constructs were transfected in HeLa cells using Lipofectamine 2000. After 24 h of transfection, fluorescent cells were imaged using an inverted fluorescence microscope (Olympus IX73), and the fluorescence was quantified by flow cytometry (CytoFLEX S, Beckman Coulter).

For dual luciferase-based assay, the ISR of *FEM1B* was cloned between the CDS of *Renilla* luciferase (RLuc) and FLuc in pcDNA3.1B. RLuc, ISR and FLuc were in same translational frame such that FLuc expression was expected only if there was SCR across the stop codon of RLuc. The negative control construct had a non-specific sequence (5′-TCCAGTGTGGTGGAATTCTGCAGATATCCA-3′) cloned in place of the ISR. In another construct, the stop codon of RLuc (UAA) was mutated to GCA, to achieve maximum FLuc activity, which was used to measure the efficiency of SCR. These constructs were transfected in HeLa cells, and the ratio of luciferase activity (FLuc/Rluc) was calculated, as explained above. These constructs were also subjected to *in vitro* transcription followed by *in vitro* translation using Rabbit Reticulocyte Lysate system (Promega). Relative luciferase activity was calculated as explained above.

### Analysis of mass spectrometry data

Mass Spectrometry raw files were downloaded from ProteomeXchange Consortium and analysed using MaxQuant Software. Variable and fixed modifications were set in MaxQuant according to the information provided along with the dataset. The list of peptides identified from a raw file was matched against 20 possible FEM1Bx protein sequences (20 different amino acids in place of the canonical stop codon) using an in-house Python script (available at https://github.com/DSqaured/Fem1B_SCR/). Peptides with score of >70 aligning, partially or fully, to the region encoded by the ISR of FEM1Bx were considered.

### Generation and characterization of anti-FEM1Bx antibody

The polyclonal antibody specific to the C-terminus extended region of FEM1Bx was generated by injecting rabbits with the synthetic peptide VTGYVKSFNVVLKSKGL (encoded in the ISR of *FEM1B*). The same peptide was used for affinity purification (Abgenex). A peptide dot blot was performed to test the specificity of this antibody. Approximately 100–200 ng of the synthetic peptide VTGYVKSFNVVLKSKGL was spotted on the methanol-activated 0.2 µm PVDF membrane (Merck Millipore). As a negative control, another synthetic peptide SSGAPVHLGQHGSQCR was used. The peptide-spotted membrane was incubated in a moist chamber for 1.5 h at room temperature. The membrane was then blocked with 5% BSA in PBS for 1 h at room temperature, and then incubated with 1:1000 anti-FEM1Bx antibody overnight. This was followed by incubation with horseradish peroxidase-conjugated secondary antibody. The blot was then developed using Clarity ECL reagent (Bio-Rad), and the images were recorded using a LAS-3000 imager (Fujifilm). For peptide blocking experiment, the anti-FEM1Bx antibody was incubated with the peptide VTGYVKSFNVVLKSKGL (1 mg/ml) overnight before using it for dot blot.

### shRNA-mediated knockdown of *FEM1B*

Exponentially growing HepG2 cells were harvested and counted using a haemocytometer. A total of 10^6^ cells were taken in Opti-MEM medium (Gibco), mixed with 4 µg of shRNA constructs (Sigma) for 10 min at room temperature. They were then transferred to an electro-cuvette and electroporated with exponential decay pulse at 160 V using Gene Pulser Xcell^TM^ Electroporation system (Bio-Rad) and seeded in a six-well plate. After 48 h of electroporation, cells were selected using puromycin (2 µg/ml. Sigma, P8833) for 7 days, and selected cells were maintained in DMEM with 10% FBS containing puromycin (0.5 µg/ml). Western blotting was performed to confirm the knockdown. *FEM1B* shRNA, TRC Clone ID:TRCN0000303647; target sequence: 5′-ACGCTTGCTCTTAGAACATTAC-3′. Control shRNA, MISSION^®^ pLKO.1-puro Non-Mammalian shRNA Control Plasmid (Sigma).

### Generation of FEM1B^X628Q−/−^ HeLa cells using the CRISPR-Cas9 system

Three sgRNA-coding sequences (5′-CAAACTCTTCAAGAGTTCTG-3′, 5′-ATATGTAAAGTCGTTTAATG-3′ and 5′-GTTGGATTTCATTAAGTGAC-3′) that target the *FEM1B* gene near its canonical stop codon were cloned in pSpCas9(BB)-2A-GFP (PX458). The sgRNA 5′-CAAACTCTTCAAGAGTTCTG-3′ was chosen based on the Inference of CRISPR Edits analysis (ICE analysis, Synthego). The repair template (∼1600 nts spanning the stop codon) with the required mutation in the canonical stop codon (UAA→CAA) was cloned into the pmCherry-C1 vector (Clontech, 632524). The plasmid expressing sgRNA and Cas9 was transfected in HeLa cells along with the plasmid expressing the repair template using Lipofectamine 2000. After 48 h, the cells positive for both GFP and mCherry were sorted and seeded at a density of one cell per well in a 96-well plate (FACSAriaTM II sorter from BD Biosciences). Individual cells were allowed to proliferate, and the colonies were screened for genome editing by PCR (primers: 5′-CCGCTAGACAAAAGTACAACTGG-3′ and 5′-TGTCCCTTGTTACATAAACAAAACA-3′) followed by restriction digestion using the EcoRI enzyme. This enzyme site is introduced only if there is UAA to CAA mutation. The mutation was then confirmed by Sanger sequencing of the PCR product.

### Dual fluorescence-based protein stability assay

DsRed and GFP fluorescence reporter construct with degron sequences were a kind gift from Itay Koren (Bar-Ilan University, Israel). They were transfected (0.5 µg/well in a 24-well plate) in wild-type or FEM1B^X628Q−/−^ or Δ3′UTR HeLa cells using polyethylenimine (Polysciences). After 24 h, the fluorescence was confirmed by imaging with an inverted fluorescence microscope (Olympus IX73). Flow cytometry (CytoFLEX S, Beckman Coulter) was carried out to quantify the fluorescence intensity. The data was analysed using CytExpert software (Beckman Coulter) to calculate the mean fluorescence intensity.

### *In vivo* tumour experiments

These experiments were approved by our Institutional Animal Ethics Committee (CAF/Ethics/862/2021). Approximately 3×10^6^ HeLa cells were inoculated subcutaneously into both the flanks of 4- to 6-week-old female athymic nude mice (Source: Central Animal Facility, Indian Institute of Science). The tumour progression was monitored using digital Vernier callipers by calculating the tumour volume [(shortest diameter)^2^×(longest diameter)×0.5].

### Sequence alignment

The nucleotide sequence of the 3′UTR of *FEM1B* mRNA from various mammalian species were taken from the NCBI database. To get corresponding amino acid sequences, the nucleotide sequences were translated computationally using the Expasy Translate tool. All sequences were aligned using Clustal Omega.

### Statistical analysis

Statistical significance was calculated using a paired or unpaired two-tailed Student's *t*-test as indicated in the figure legends. When the samples showed differences in the variance, Welch's correction was applied. A two-way ANOVA test with no post test was used to calculate statistical significance for experiments involving multiple time-points (proliferation, protein stability assays and *in vivo* tumour studies).

## Supplementary Material



10.1242/joces.261921_sup1 Supplementary information
