# Supplementary material for: Hominini-specific regulation of the cell cycle by stop codon readthrough of FEM1B
Source: J Cell Sci. 2024 Aug 29;137(16):jcs261921. doi: 10.1242/jcs.261921 (PMC11385324; doi:10.1242/jcs.261921)
Supplement: Supplementary information [file joces-137-261921-s1.pdf]

Figure S1

```
Hs : TAAGTGACTGGATATGTAAAGTCGTTTAATGTGGTGCTAAAAAGTAAAGGACTTTTAATCACAGACAGT  69
Pp : TAAGTGACTGGATATGTAAAGTCGTTTAATGTGGTGCTAAAAAGTAAAGGACTTTTAATCACAGACAGT  69
Pt : TAAGTGACTGGATATGTAAAGTCGTTTAATGTGGTGCTAAAAAGTAAAGGACTTTTAATCACAGACAGT  69
Pa : TAAGTGACTGGATATGTAAAGTCGTTTAATGTGGTGCTAAAAAGTAAAGGAC-TTTAATCACAGACAGT  68
Nl : TAAGTGACTGGATATGTAAAGTCGTTTAATGTGGTGCTAAAAAGTAAAGGAC-TTTAATCACAGACAGT  68
Pp : TAAGTGACTGGATATGTAAAATCGTTTAATGTGGTGCTAAAAAGTAAAGGAC-TTTAATCACAGACAAT  68
Fc : TAAGTGACTGGATATGTAAAATCGTTTAATGTGGTGCTAAAAAGTAAAGGAC-TTTAATCACAGACAAT  68
Oa : TAAGTGACTGGATATGTAAAATCGTTTAATGTGGTGCTAAAAAGTAAAGGAC-TTTAATCACAGACAAT  68
Ss : TAAGTGACTGGATATGTAAAATCGTTTAATGTGGTGCTAAAAAGTAAAGGAC-TTTAATCACAGACAAT  68
Pk : TAAGTGACTGGATATGTAAAATTGTTTAATGTGGTGCTAAAAAGTAAAGAAC-TTTAATCACAGACAAT  68
Mm : TAAGTGACTGGATGTGTAAAATCGTTTAATGTGGTGCTAAAAAGTAAAGGAC-TTTAATCACAGACAAT  68
     ************* ****** * ***************************** ************** *

Hs : AGAATTATGTGTTCATAAATTCTGCTTTTCTTTCCACTACCCTTCCTCCCATCCCATCCTTCCTTAGTT  138
Pp : AGAATTATGTGTTCATAAATTCTGCTTTTCTTTCCACTACCCTTCCTCCCATCCCATCCTTCCTTAGTT  138
Pt : AGAATTATGTGTTCATAAATTCTGCTTTTCTTTCCACTACCCTTCCTCCCATCCCATCCTTCCTTAGTT  138
Pa : AGAATTATGTGTTCATAAATTCTGCTTTTCTTTCCACTACCCTTCCTCCCATCCCATCCTTCCTTAGTT  137
Nl : AGAATTATGTGTTCATAAATTCTGCTTTTCTTTCCACTACCCTTCCTCCCATCCCATCCTTCCTTAGTT  137
Pp : AGAATTATGTGTTCATAAATTCTGCTTTTCTTTCCATTACCCTTCCTCCCTACCCATCCTTCCTTAGTT  137
Fc : AGAATTATGTGTTCATAAATTCTGCTTTTCTTTCCATTACCCTTCTTCCCTATCCATCCTTCCTTAGTT  137
Oa : AGAATTATGTGTTCATAAATTCTACTTTTCTTTCCACTACCCCTCCTCCCTATCCTTCCTTAGTTTTGT  137
Ss : AGAATTATGTGTTCATAAATTCTACTTTTCTTTCCACTACTCTTCCTCCCTACCCAGCCTTCCTTAGTT  137
Pk : AGAATTATGTGTTCATAAATTCTACTTTTCTTTCCATTACCCTTTCTCTATACCCACCCTTCCTTAGTT  137
Mm : AGAATTCTGTGTTTGTAAATTCTCCTTTTCTTTCCACTATCCTTTCAGCCATCGTCCTTGGTTCCGTGT  137
     ****** ******  ******** ************ **  * *                        *

Hs : CTGTATTTGTTTTTCTTGCCTCATGGTAATTG---ATTTCAGACAGACTTTAACAAA-ACCACATTGTT  203
Pp : CTGTATTTGTTTTTCTTGCCTCATGGTAATTG---ATTTCAGACAGACTTTAACAAA-ACCACATTGTT  203
Pt : CTGTATTTGTTTTTCTTGCCTCATGGTACTTG---ATTTCAGACAGACTTTAACAAA-ACCACATTGTT  203
Pa : CTGTATTTGTTTTTCTTGCCTCATGATAATTG---ATTTCAGACAGACTTTAAACAA-AGCCACATAGT  202
Nl : CTCTATTTGTTTTTCTTGCCTCATGATAATTG---ATTTCAGACAGACTTTAAACAA-AGCCACATTGT  202
Pp : CTGTATTTGGTCTTTTTGCCTCATGACGTTGACTGATCTCAAATACACTTTAAACAACGCCACATTGTT  206
Fc : CTGTATTTGGTCTTTTTGCCTCATGATGTTGACTGATCTCAAATACACTTTAAACAACGCGACATTGTT  206
Oa : TTGTATTTGGTCTTCTTGTCTCATGTGGTTAT-TGATTTCAAATACACTTTAAACAAAGCCACATTGTT  205
Ss : TTGTATGTGGTCTTCTTGCCTCATATGGTAAT-TGATTTCAAATATACTTTAAACAAAGCCACATTGTT  205
Pk : TTGTATTTGGTCTTCTTGCCTCATATGGTAAT-TGATTTCAAATACACTTTAAACAAAGCCACATTGTT  205
Mm : GCCTCTTTTCTCACGTGGTATTGAGCTCA----------------GCACACTAAACAGAGCAGCGCTGTT  191
        * * *  *    * *  *                         **  ***  *            *
```

Fig. S1. Proximal 3'UTR of *FEM1B* mRNA is evolutionarily conserved.

Alignment of nucleotide sequences of the proximal 3'UTR of *FEM1B* from 11 mammalian species. In-frame stop codons are shown in red. Conserved nucleotides are indicated with asterisks. Arrow points to 53rd nucleotide where a 'T' is inserted in the sequences of humans and chimpanzees. Hs, *Homo sapiens*; Pp, *Pan paniscus*; Pt, *Pan troglodytes*; Pa, *Papio anubis*; Nl, *Nomascus leucogenys*; Pp, *Panthera pardus*; Fc, *Felis catus*; Oa, *Ovis aries*; Ss, *Sus scrofa*; Pk, *Pipistrellus kuhlii*; Mm, *Mus musculus*.

Journal of Cell Science • Supplementary information

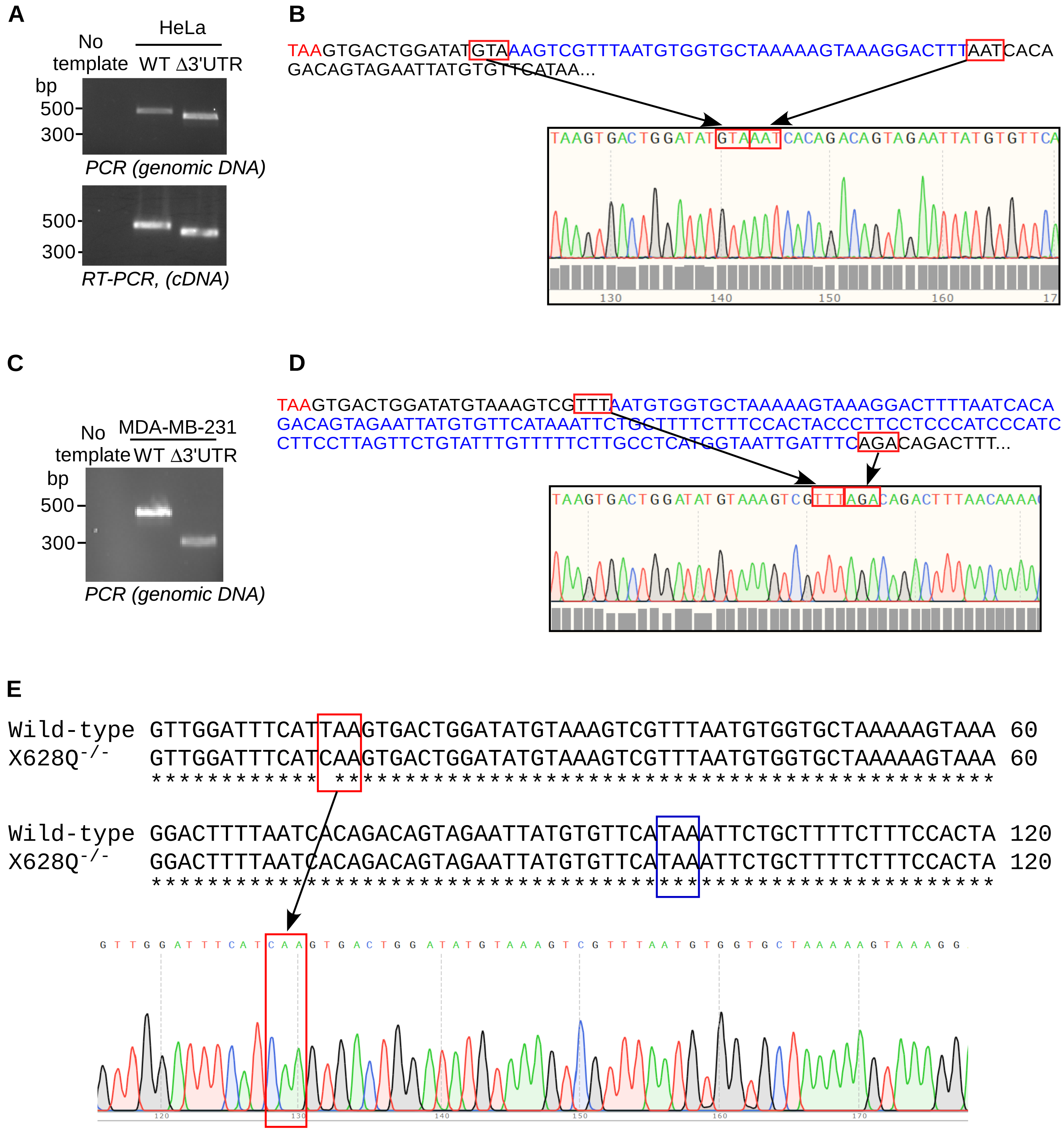

Fig. S2. Confirmation of genome edited cells generated by CRISPR-Cas9 system.
(A) Results of *FEM1B* PCR performed using genomic DNA or cDNA from wild-type or Δ3'UTR HeLa cells.
(B) Electropherogram peaks of Sanger sequencing of the product of *FEM1B* PCR performed using genomic DNA of Δ3'UTR HeLa cells. The deleted region is shown in blue.
(C) Results of *FEM1B* PCR performed using genomic DNA from wild-type or Δ3'UTR MDA-MB- 231 cells.
(D) Electropherogram peaks of Sanger sequencing of the product of *FEM1B* PCR performed using genomic DNA of Δ3'UTR MDA-MB-231 cells. The deleted region is shown in blue.
(E) Alignment of the sequence around the canonical stop codon of *FEM1B* gene from FEM1B$^{X628Q-/-}$ cells and the parental wild-type cells. In these cells the canonical stop codon, TAA, was mutated to CAA using CRISPR-Cas9 system. Electropherogram peaks of Sanger sequencing of the product of *FEM1B* PCR performed using genomic DNA of FEM1B$^{X628Q-/-}$HeLa cells. Location of the mutated nucleotide in the canonical stop codon (TAA to CAA) is shown in a red box. The downstream in-frame stop codon is highlighted in a blue box.

Journal of Cell Science • Supplementary information

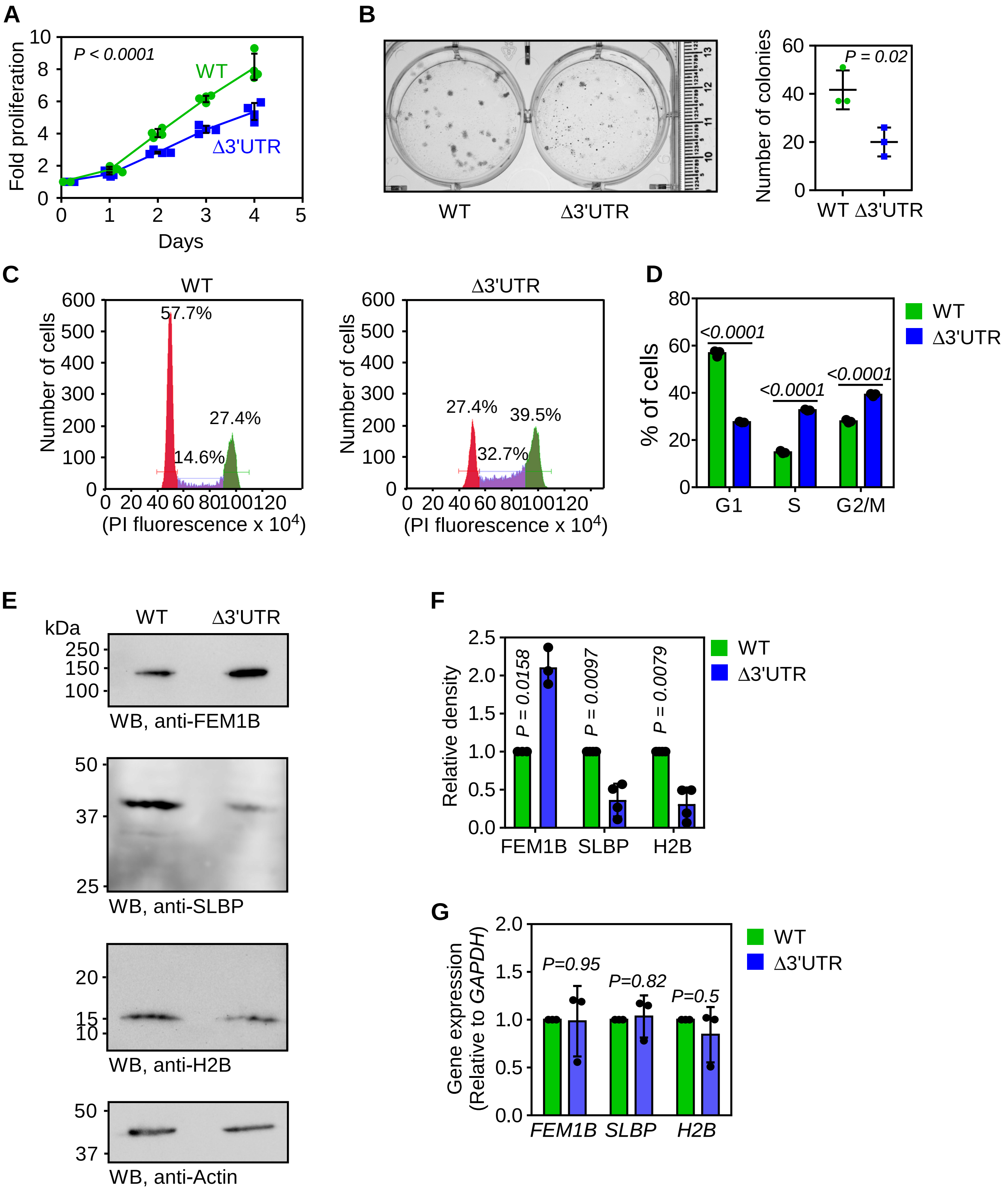
A
P < 0.0001
WT
Δ3'UTR
Fold proliferation
Days
B
WT
Δ3'UTR
Number of colonies
P = 0.02
C
WT
Δ3'UTR
Number of cells
57.7%
14.6%
27.4%
27.4%
32.7%
39.5%
(PI fluorescence x 10^4)
D
% of cells
<0.0001
G1
S
G2/M
E
kDa
WB, anti-FEM1B
WB, anti-SLBP
WB, anti-H2B
WB, anti-Actin
F
Relative density
P = 0.0158
P = 0.0097
P = 0.0079
FEM1B
SLBP
H2B
G
Gene expression
(Relative to GAPDH)
P=0.95
P=0.82
P=0.5

Fig. S3. Δ3'UTR MDA-MB-231 cells show cell cycle delay and enhanced FEM1B expression.
(A) Cell proliferation assay performed using MTT in wild-type and Δ3'UTR MDA-MB-231 cells. Lines join the mean values of fold proliferation at 1st to 4th day relative to 0th day (N=3 biological replicates; error bars, SD; *P* value, 2-way ANOVA).
(B) Representative images of clonogenicity assay (left). Quantification of colonies is shown in the graph (Mean ± SD; N=3 biological replicates; *P* value, two-tailed Student's t-test).
(C) Cell cycle analyses performed 30 h after cells were released from the double thymidine block. Propidium iodide treated cells were analysed using a flow cytometer. % of cells belonging to G1 (red), S (purple), and G2/M (green) phases of cell cycle are shown.
(D) The graph shows the quantification of the cell cycle analyses (Mean ± SD; N=3 biological replicates; *P* value, two-tailed Student's t-test).
(E) Western blot showing the cellular levels of FEM1B (dimer), SLBP and H2B in wild-type and Δ3'UTR MDA-MB-231 cells.
(F) The graph shows the densitometry values (mean ± SD; N=3 or 4 biological replicates; *P* value, two-sided paired t-test).
(G) Expression of *FEM1B*, *SLBP* and *H2B* relative to *GAPDH* calculated by quantitative real-time PCR in wild-type and Δ3'UTR MDA-MB-231 cells. Graph shows mean ± SD (N=3 biological replicates; *P* value, two-sided paired t-test).

Journal of Cell Science • Supplementary information

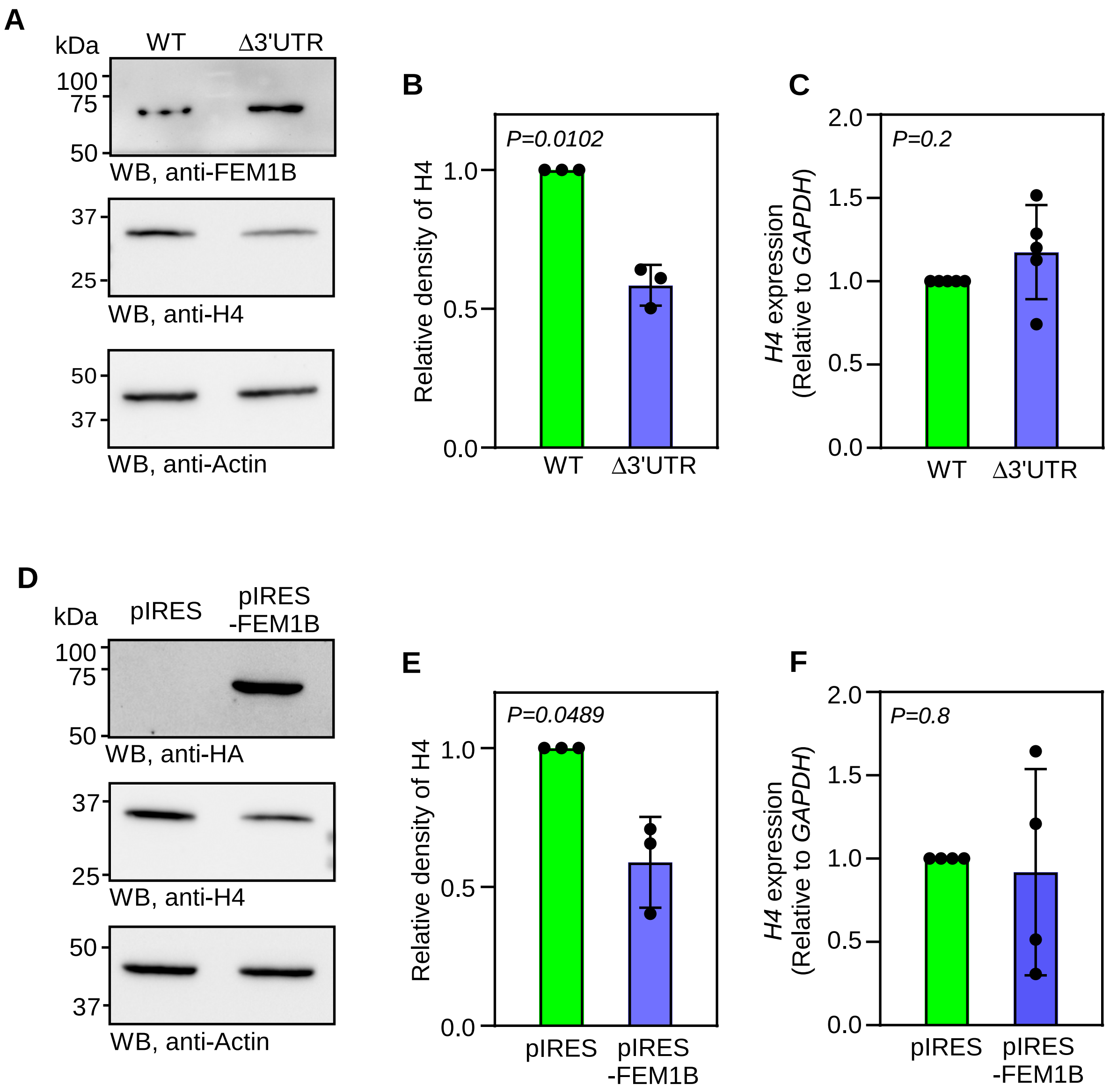

Fig. S4. Cellular levels of FEM1B regulates H4 expression.

(A) Western blot showing the cellular levels of FEM1B and H4 in wild-type and Δ3'UTR HeLa cells.(B) The graph shows the densitometry values (mean ± SD, N=3 biological replicates).

(C) Expression of *H4* relative to GAPDH calculated by quantitative real-time PCR in wild-type and Δ3'UTR cells (mean ± SD, N=5 biological replicates).

(D) Western blot showing the cellular levels of FEM1B and H4 in HeLa cells stably overexpressing HA-tagged FEM1B.

(E) The graph shows the densitometry values (mean ± SD, N=3 biological replicates).

(F) Expression of *H4* relative to GAPDH calculated by quantitative real-time PCR in HeLa cells stably overexpressing HA-tagged FEM1B. (mean ± SD, N=4 biological replicates)

Statistical significance in all graphs was calculated by two-tailed paired t-test.

Journal of Cell Science • Supplementary information

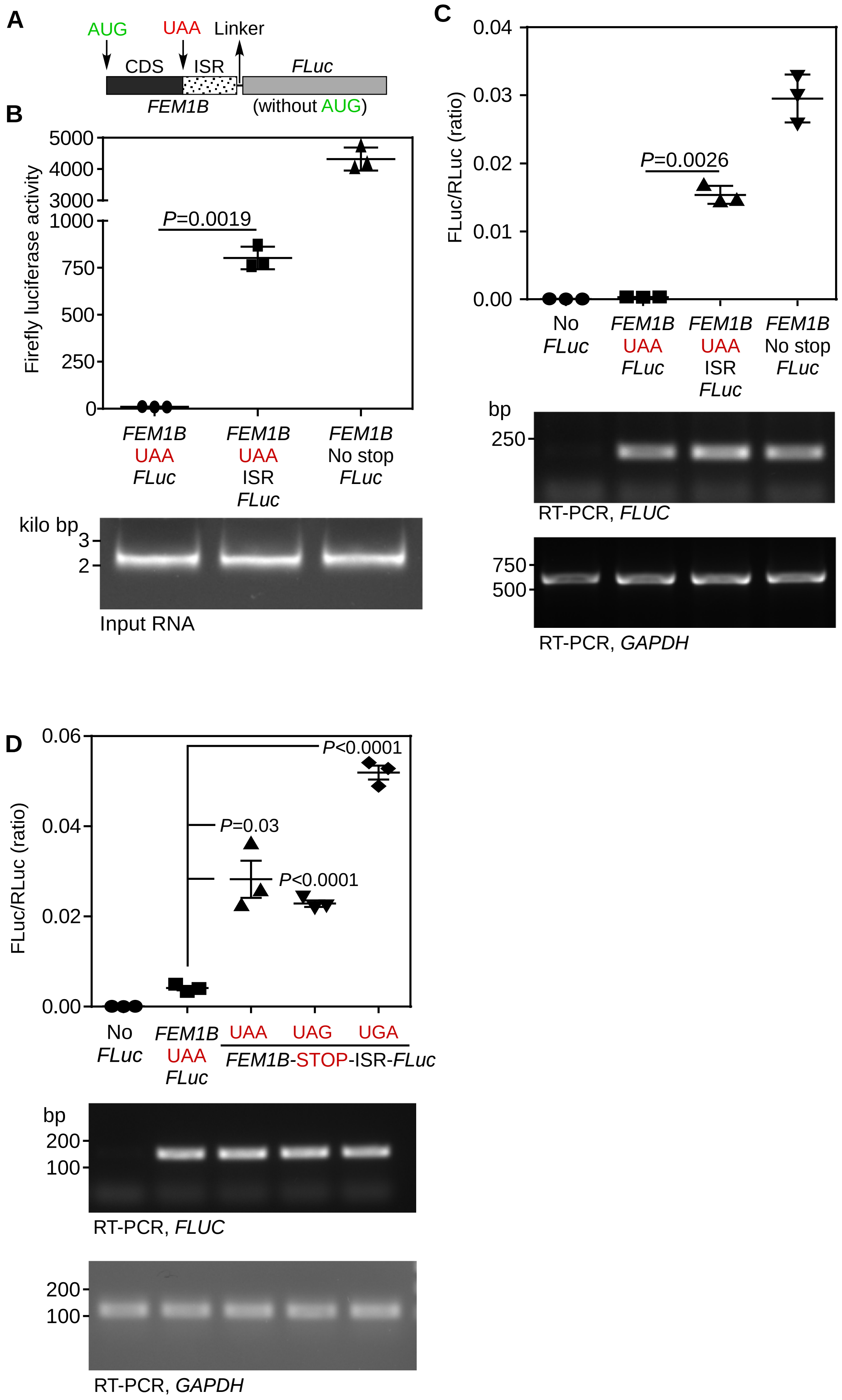

Journal of Cell Science • Supplementary information

Fig. S5. Demonstration of SCR of *FEM1B* by luminescence-based assay.

(A) Schematic of the plasmid constructs used in luminescence-based SCR assay (FLuc, firefly luciferase).

(B) Results of luminescence-based SCR assay performed *in vitro* using rabbit reticulocyte lysate (mean ± SD; N=3 biological replicates). SCR activity is indicated by firefly luciferase activity (luminescence). Equal amounts of *in vitro* transcribed RNAs were used for this assay (bottom).

(C) Results of luminescence-based SCR assay performed in HeLa cells. SCR activity is indicated as activity of firefly luciferase relative to that of co-transfected Renilla luciferase (FLuc/RLuc mean ± SD; N=3 biological replicates). RT-PCR analysis shows FLuc mRNA levels in these cells.

(D) The effect of stop codon mutation on SCR. The SCR assay was performed as in (C). The UAA stop codon of the construct shown in (A) was mutated to UAG and UGA for the assay. SCR activity is indicated as activity of firefly luciferase relative to that of co-transfected Renilla luciferase (FLuc/RLuc mean ± SD; N=3 biological replicates). RT-PCR analysis shows FLuc mRNA levels in these cells.

P values were calculated by two-tailed Student’s t-test.

Journal of Cell Science • Supplementary information

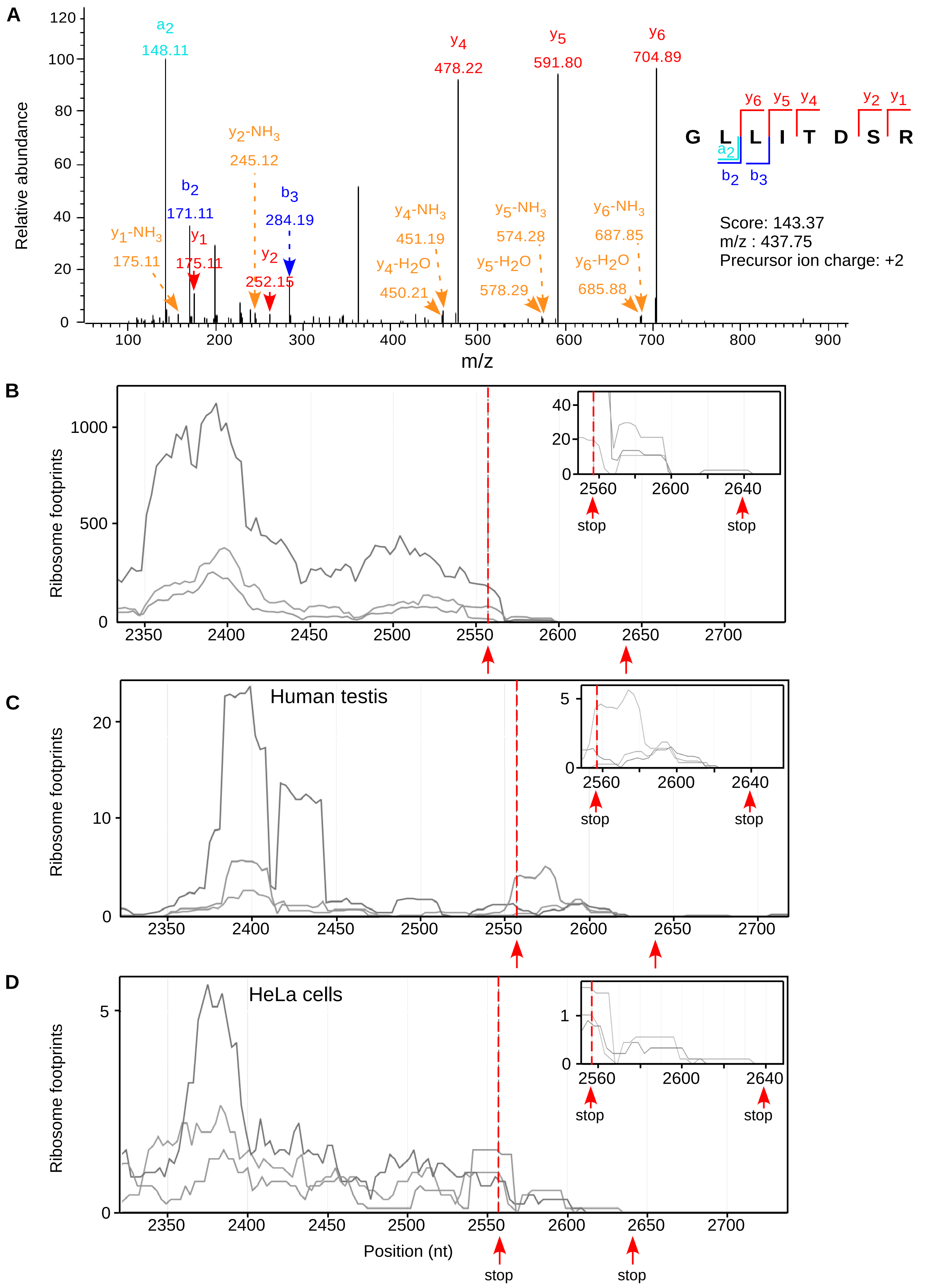

Journal of Cell Science • Supplementary information

Fig. S6. Direct evidence for SCR.

(A) Mass spectrometry: Spectra of GLLITDSR, a peptide unique to the ISR of *FEM1B*, identified in the mass spectrometry data derived from primary human testicular peritubular cells (source of the data: PXD033534). The analysis was performed using MaxQuant, a quantitative proteomics software. (B, C and D) Ribosome profiling data analysis: The analysis was performed using the ribosome profiling data available in RiboCrypt (https://ribocrypt.org/). Y-axis represents the number of ribosome footprints on *FEM1B* mRNA (Transcript ID: ENST00000306917) from (B) all ribosome profiling samples available in the online tool (all_merged-Homo_sapiens), (C) the project PRJEB28810 (Source: testis from *Homo sapiens;* library: RFP_testis_r2) and (D) the project PRJNA218051 (Source: HeLa cells; library: RFP_S_r2). Three lines represent footprints in three translational frames. X-axis show the position of nucleotides of *FEM1B* mRNA. Region between 2350 and 2700 nucleotides is shown. The ISR (inter stop codon region) is enlarged in the inset image. Red arrows indicate the position of two stop codons.

Journal of Cell Science • Supplementary information

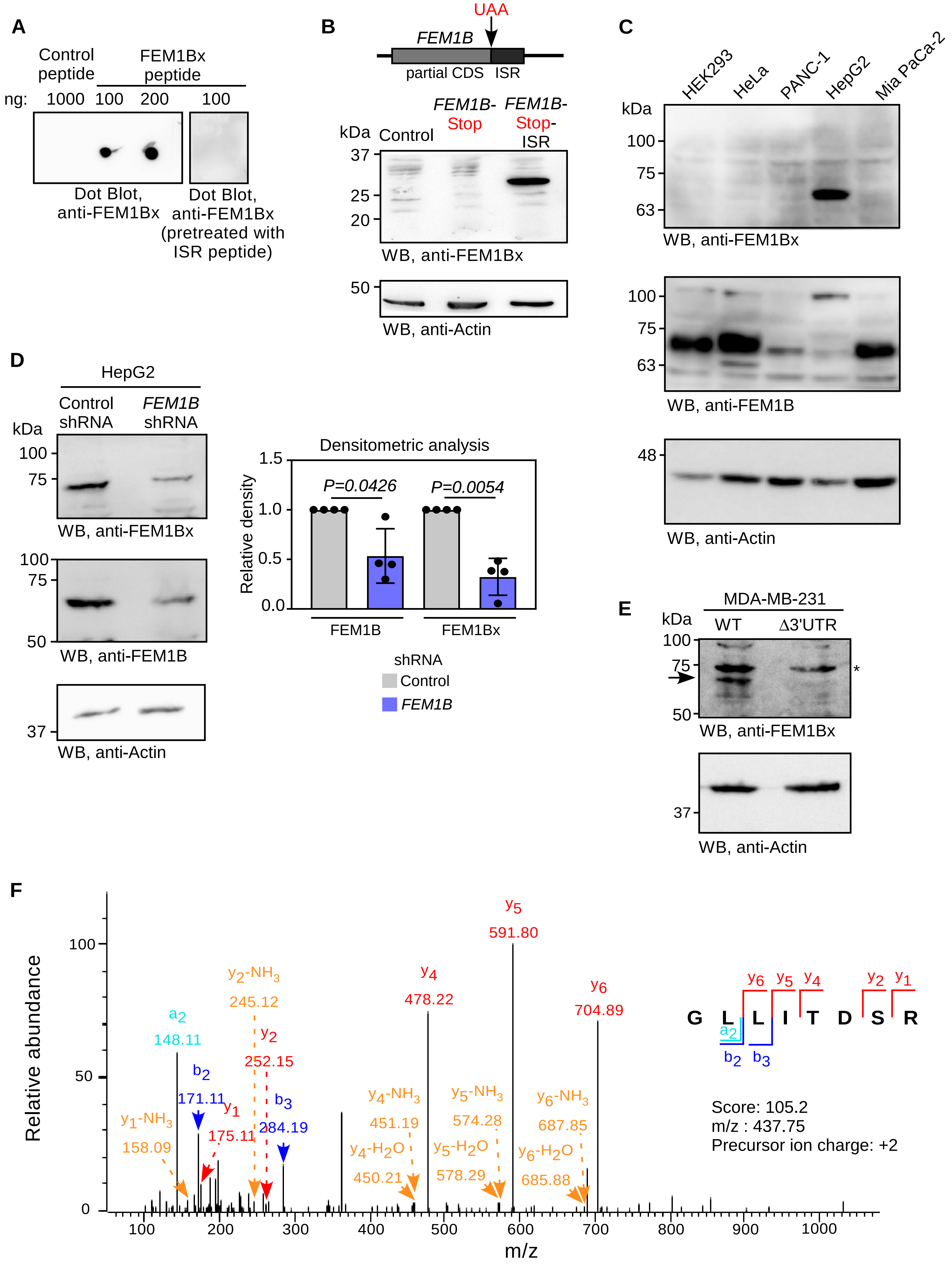
A
Control peptide
FEM1Bx peptide
ng: 1000 100 200 100
Dot Blot, anti-FEM1Bx
Dot Blot, anti-FEM1Bx (pretreated with ISR peptide)
B
UAA
FEM1B
partial CDS ISR
kDa Control FEM1B-Stop FEM1B-Stop-ISR
37
25
20
WB, anti-FEM1Bx
50
WB, anti-Actin
C
HEK293 HeLa PANC-1 HepG2 Mia PaCa-2
kDa
100
75
63
WB, anti-FEM1Bx
WB, anti-FEM1B
48
WB, anti-Actin
D
HepG2
Control shRNA
FEM1B shRNA
kDa
WB, anti-FEM1Bx
WB, anti-FEM1B
37
WB, anti-Actin
Densitometric analysis
P=0.0426
P=0.0054
Relative density
FEM1B FEM1Bx
shRNA
Control
FEM1B
E
MDA-MB-231
kDa WT Δ3'UTR
WB, anti-FEM1Bx
WB, anti-Actin
F
Relative abundance
m/z
G L L I T D S R
Score: 105.2
m/z : 437.75
Precursor ion charge: +2

Fig. S7. Detection of endogenous SCR product.

(A) Dot blot assay using anti-FEM1Bx antibody. The assay was performed using the synthetic peptide encoded by *FEM1B* ISR and a control peptide as described in Methods.

(B) Western blot showing the expression of the SCR product of *FEM1B* in HEK293 cells transfected with the construct shown in the schematic. Anti-FEM1Bx antibody was used to detect the SCR product.

(C) Western blot showing the expression of the SCR product of endogenous *FEM1B* (FEM1Bx) in multiple cell lines.

(D) Western blots showing the expression of endogenous FEM1B and FEM1Bx in HepG2 cells expressing a *FEM1B*-targeting shRNA. FEM1B was detectable after prolonged exposure compared to FEM1Bx. The graph shows the densitometric analysis (Mean ± SD, N=4 biological replicates; *P* values, two-tailed paired t-test).

(E) Western blot showing the expression of endogenous FEM1Bx in wild-type and Δ3'UTR MDA-MB-231 cells. Arrow indicates FEM1Bx and * indicates a nonspecific band.

(F) Mass spectrometry: Spectrum of GLLITDSR, a peptide unique to the ISR of *FEM1B*, identified in the mass spectrometry data derived from HepG2 cells (source of the data: PXD029268). The analysis was performed using MaxQuant, a quantitative proteomics software.

Journal of Cell Science • Supplementary information

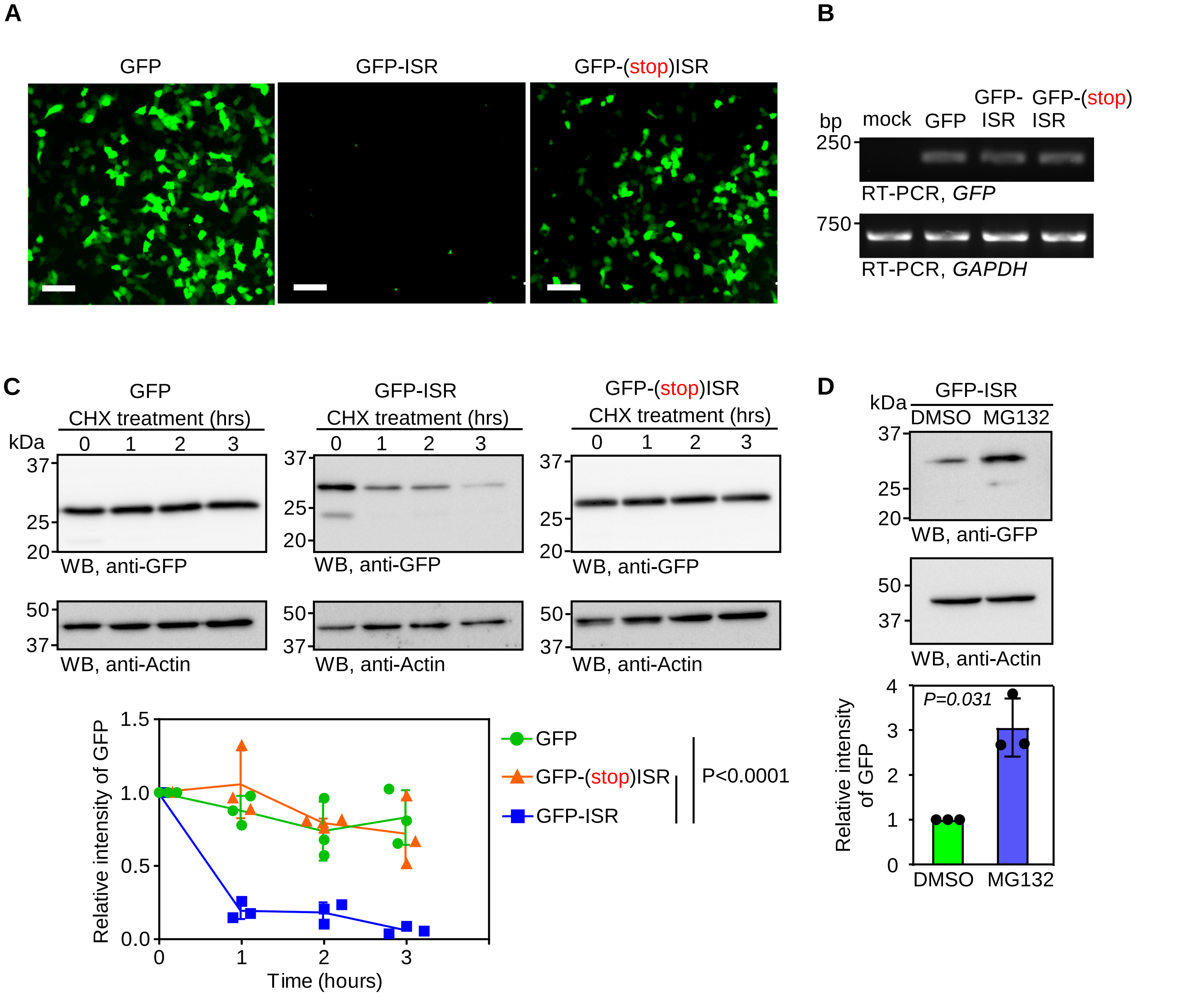

Fig. S8. The peptide encoded by the ISR of *FEM1B* is sufficient to induce protein degradation. (A) Fluorescence microscopy images of HeLa cells expressing GFP and GFP with the ISR of *FEM1B*. Scale bar, 100 µm.

(B) RT-PCR results showing the expression of GFP in these cells.

(C) Western blots showing the levels of GFP, GFP-ISR and GFP-stop-ISR in HeLa cells treated with cycloheximide (100 µg/mL) for 1, 2, and 3 h.

(D) Western blot showing the expression of GFP-ISR in HeLa cells treated with MG132 (10 µM for 6 h). The graphs show the mean density of the bands ± SD (N=3 biological replicates).

P values were calculated by 2-way ANOVA test (C) or two-tailed Student's t-test (D).

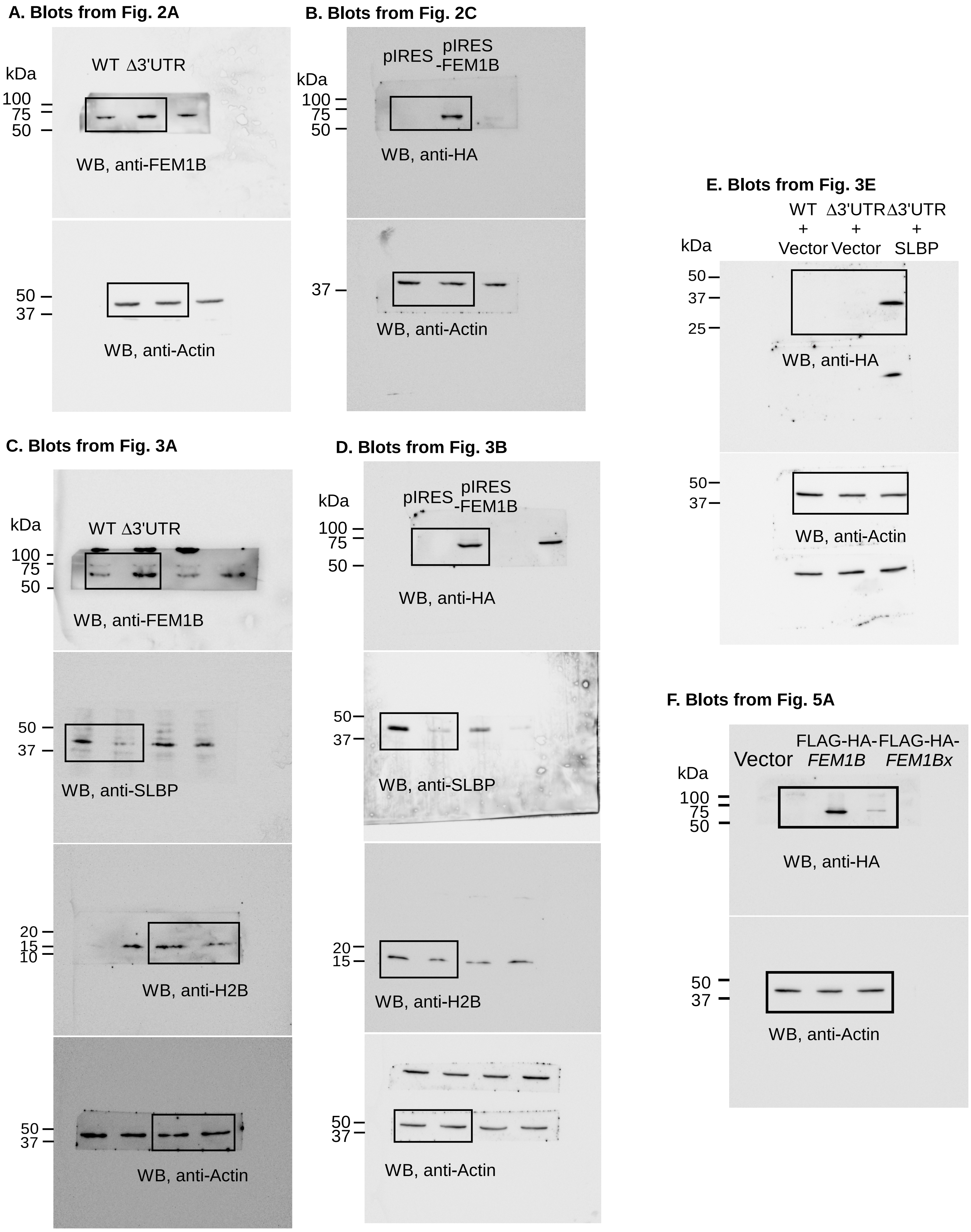

Blot transparency

Journal of Cell Science • Supplementary information

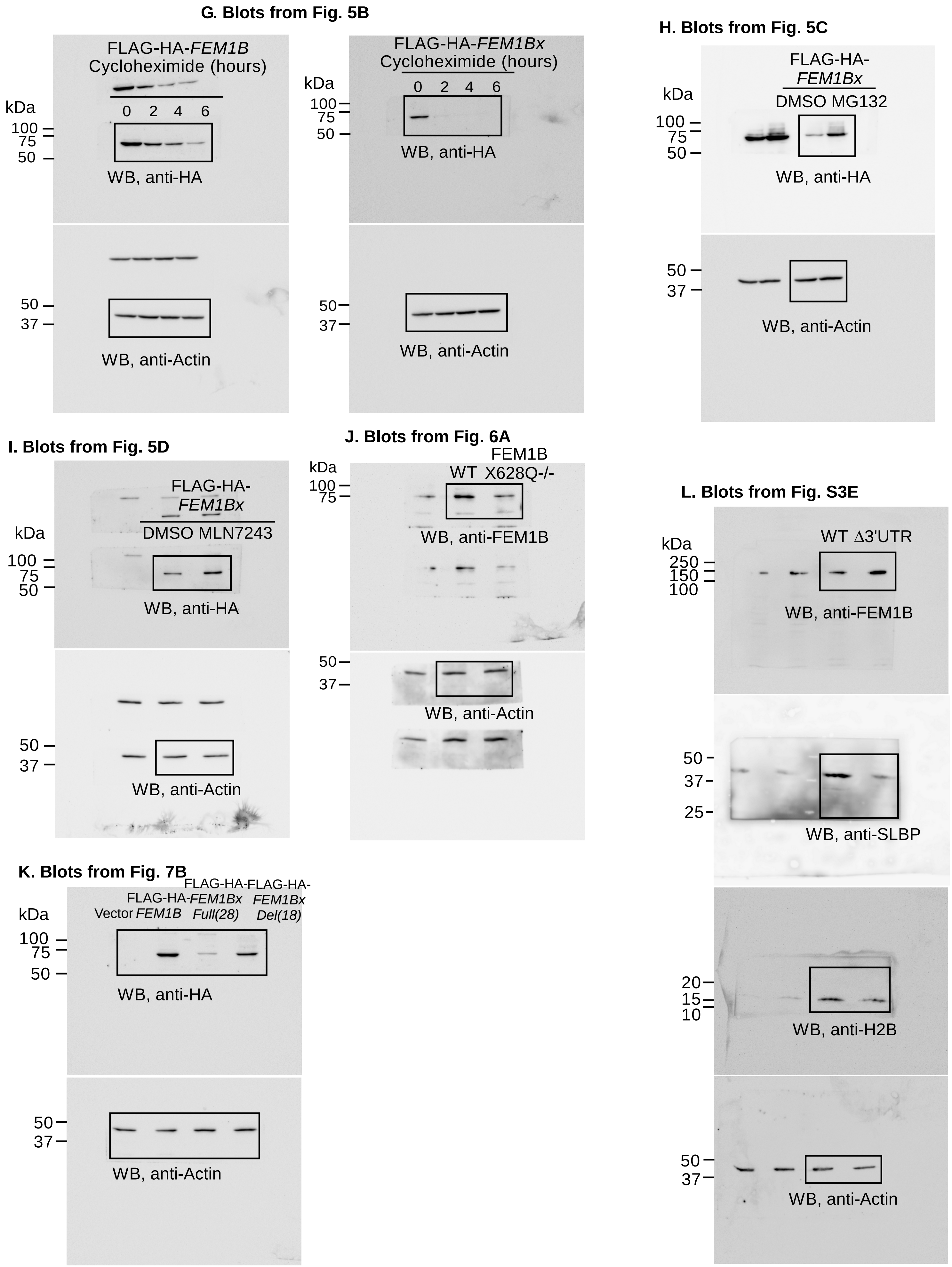

Blot transparency

Journal of Cell Science • Supplementary information

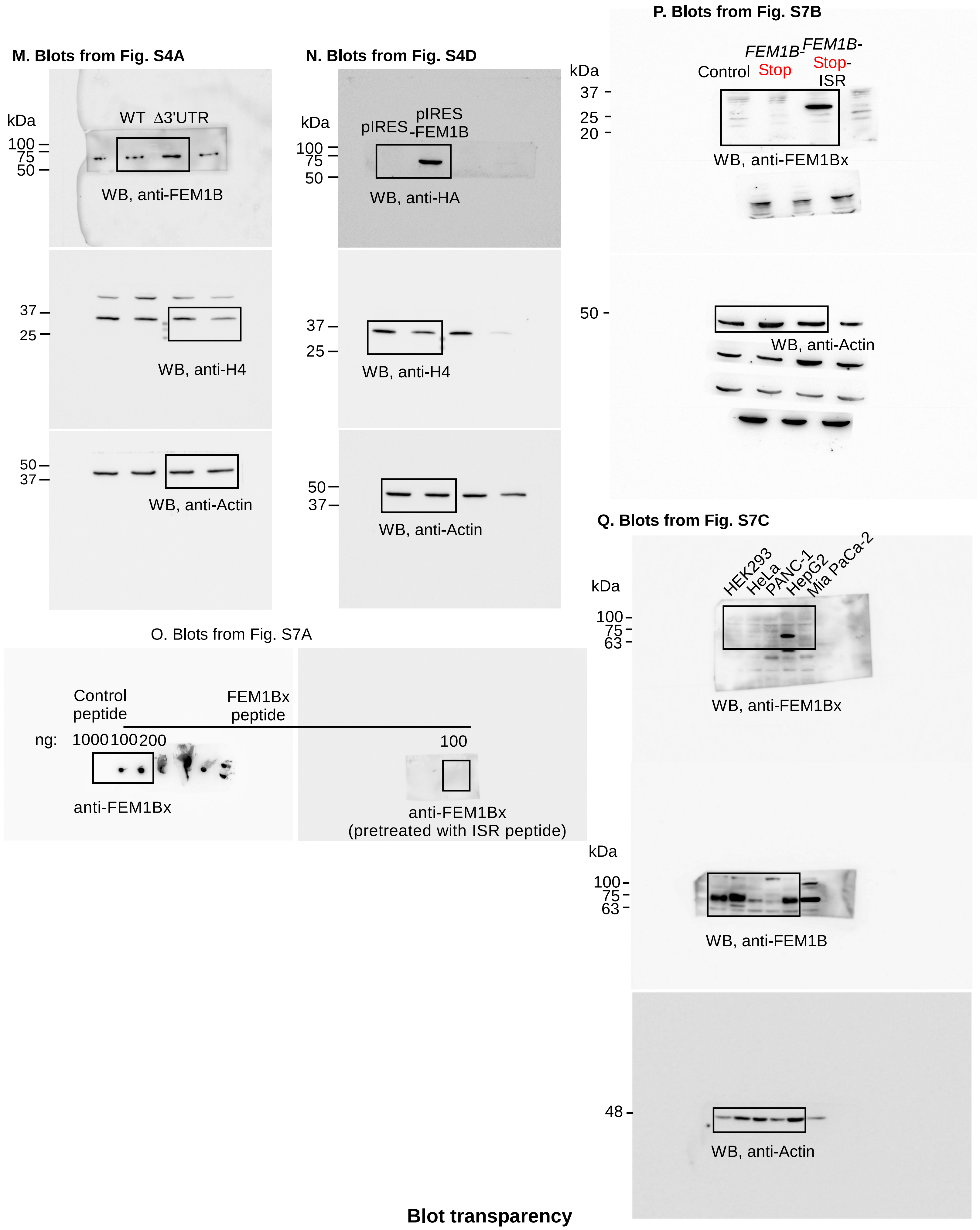

Blot transparency

Journal of Cell Science • Supplementary information

R. Blots from Fig. S7D

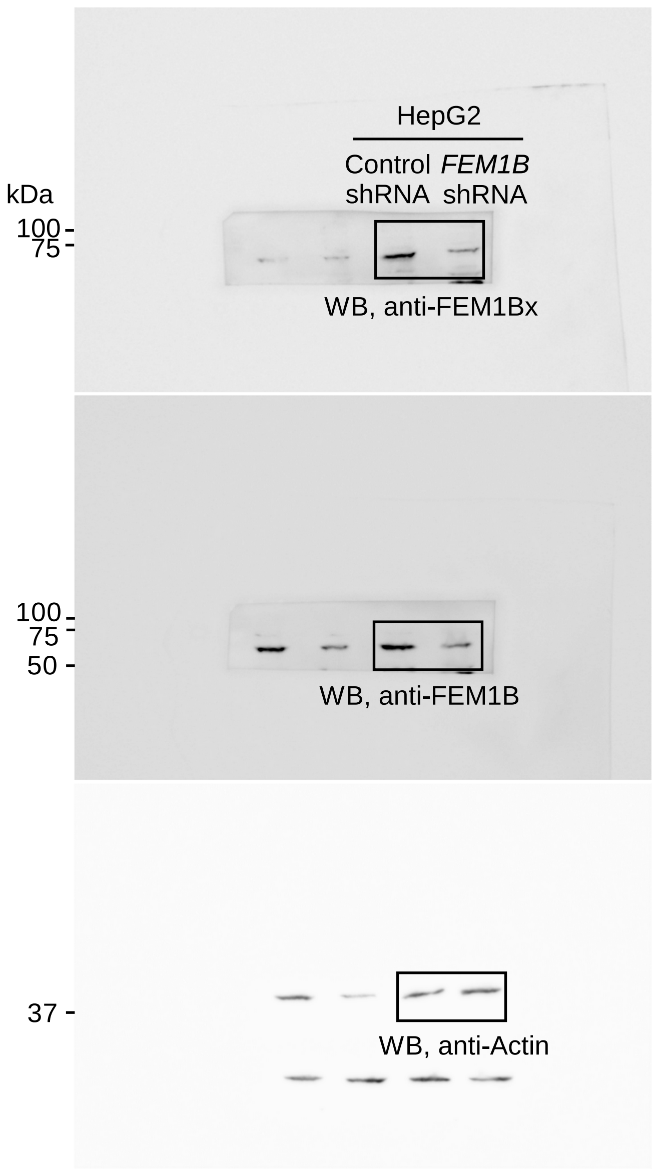

S. Blots from Fig. S7E

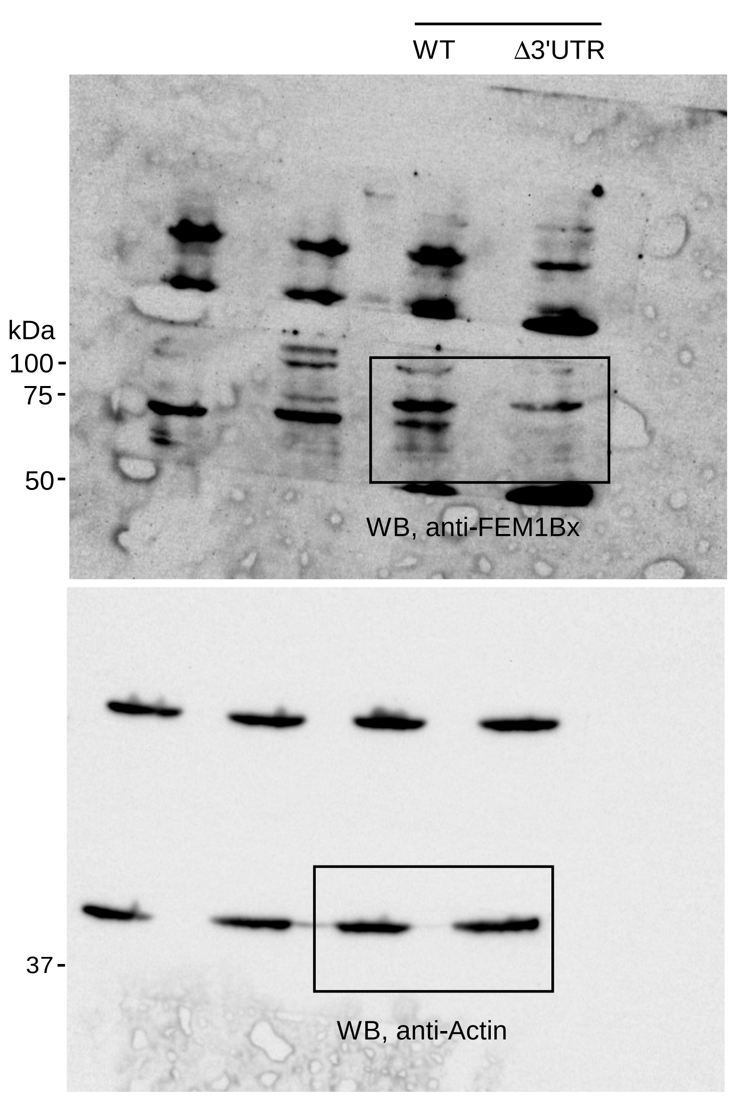

T. Blots from Fig. S8C

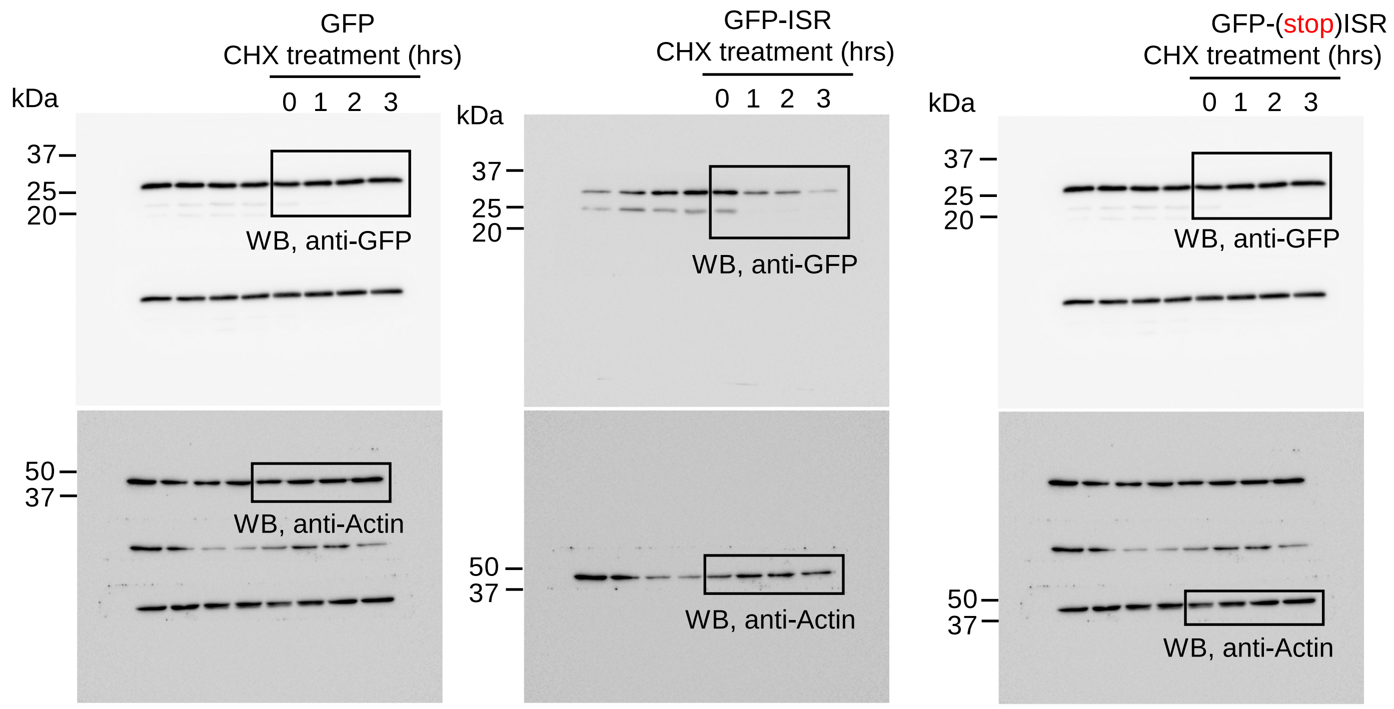

Blot transparency

Journal of Cell Science • Supplementary information

U. Blots from Fig. S8D

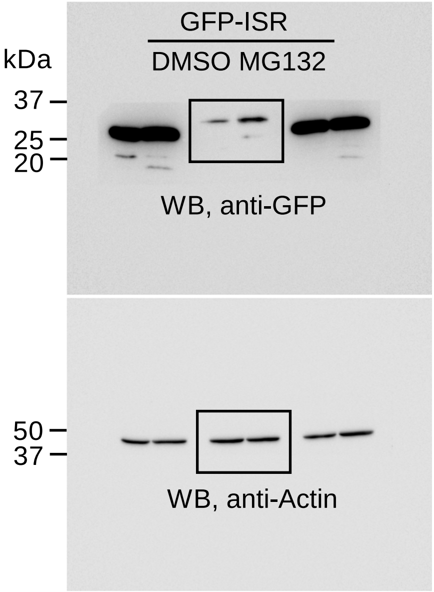

Fig. S9. Blot transparency

Uncropped images of western blots are shown in the following figure panels:

A. Blots from Fig. 2A
B. Blots from Fig. 2C
C. Blots from Fig. 3A
D. Blots from Fig. 3B
E. Blots from Fig. 3E
F. Blots from Fig. 5A
G. Blots from Fig. 5B
H. Blots from Fig. 5C
I. Blots from Fig. 5D
J. Blots from Fig. 6A
K. Blots from Fig. 7B
L. Blots from Fig. S3E
M. Blots from Fig. S4A
N. Blots from Fig. S4D
O. Blots from Fig. S7A
P. Blots from Fig. S7B
Q. Blots from Fig. S7C
R. Blots from Fig. S7D
S. Blots from Fig. S7E
T. Blots from Fig. S8C
U. Blots from Fig. S8D

Journal of Cell Science • Supplementary information

Table S1. Luminescence values for Fig 4C (HeLa)

Samples	FLuc	RLuc
RLuc UAA non sp. *FLuc*	134	8110
	111	8630
	84	4630
RLuc UAA ISR *FLuc*	778	9370
	592	8050
	663	8880
RLuc GCA ISR *FLuc*	4650	9790
	4520	10600
	4380	10200

Table S2. Luminescence values for Fig 4C (*In vitro*)

Samples	FLuc	RLuc
RLuc UAA non sp. *FLuc*	8572	3303000
	7570	3099000
	7502	3072000
RLuc UAA ISR *FLuc*	67070	3931000
	67930	3665000
	69730	3494000
RLuc GCA ISR *FLuc*	80040	1470000
	81570	1586000
	81980	1469000

Table S3. Luminescence values for Fig. 7C

Samples	FLuc	RLuc
RLuc UAA non sp. *FLuc*	171	10400
	194	13000
	160	8780
RLuc UAA ISR *(hs81) FLuc*	802	10700
	723	11000
	890	10900
RLuc UAA ISR *(hs51) FLuc*	231	14600
	217	14800
	205	12900
RLuc UAA ISR *(mm51) FLuc*	265	13600
	300	13800
	260	14500
RLuc GCA ISR *(hs81) FLuc*	4630	9390
	4680	9700
	4350	9260

Table S4. Luminescence values for Fig. S5C

Samples	FLuc	RLuc
No *FLuc*	148	2370000
	145	2030000
	109	2200000
FEM1B UAA *FLuc*	368	1100000
	347	1110000
	347	1040000
FEM1B UAA ISR *FLuc*	16200	1120000
	18600	1100000
	18200	1240000
FEM1B No stop *FLuc*	56700	1890000
	59100	2290000
	54700	1670000

Table S5. Luminescence values for Fig. S5D

Samples	FLuc	RLuc
No *FLuc*	284	3280000
	184	3020000
	146	3130000
FEM1B UAA *FLuc*	5610	1670000
	5470	1360000
	6270	1260000
FEM1B UAA ISR *FLuc*	35200	1360000
	31700	1400000
	40700	1120000
FEM1B UAG ISR *FLuc*	26500	1090000
	27400	1250000
	27500	1230000
FEM1B UGA ISR *FLuc*	31200	591000
	37300	690000
	36600	749000

Journal of Cell Science • Supplementary information
