# Supplementary material for: Hominini-specific regulation of the cell cycle by stop codon readthrough of FEM1B
Source: J Cell Sci. 2024 Aug 29;137(16):jcs261921. doi: 10.1242/jcs.261921 (PMC11385324; doi:10.1242/jcs.261921)
Supplement: Supplementary file 1 [file joces-137-261921_review_history.pdf]

Hominini-specific regulation of the cell cycle by stop codon readthrough of *FEM1B*

Md Noor Akhtar, Anumeha Singh, Lekha E. Manjunath, Dhruba Dey, Sangeetha Devi Kumar, Kirtana Vasu, Arpan Das and Sandeep M. Eswarappa
DOI: 10.1242/jcs.261921

Editor: Renata Basto

Review timeline

Original submission:	21 December 2023
Editorial decision:	7 February 2024
First revision received:	2 April 2024
Editorial decision:	8 May 2024
Second revision received:	5 July 2024
Accepted:	25 July 2024

Original submission

First decision letter

MS ID#: JOCES/2023/261921

MS TITLE: Hominini-specific regulation of cell cycle by stop codon readthrough of FEM1B

AUTHORS: Md Noor Akhtar, Anumeha Singh, Lekha Manjunath, Dhruba Dey, Sangeetha Devi Kumar, Kirtana Vasu, Arpan Das, and Sandeep Eswarappa

We have now reached a decision on the above manuscript.

To see the reviewers' reports and a copy of this decision letter, please go to: https://submit-jcs.biologists.org and click on the 'Manuscripts with Decisions' queue in the Author Area. (Corresponding author only has access to reviews.)

As you will see, reviewer 1 raise a number of substantial criticisms that prevent me from accepting the paper at this stage. They suggest, however, that a revised version might prove acceptable, if you can address their concerns. If you think that you can deal satisfactorily with the criticisms on revision, I would be pleased to see a revised manuscript. We would then return it to the reviewers.

Please ensure that you clearly highlight all changes made in the revised manuscript. Please avoid using 'Tracked changes' in Word files as these are lost in PDF conversion.

I should be grateful if you would also provide a point-by-point response detailing how you have dealt with the points raised by the reviewers in the 'Response to Reviewers' box. Please attend to all of the reviewers' comments. If you do not agree with any of their criticisms or suggestions please explain clearly why this is so.

Reviewer 1

Advance summary and potential significance to field

© 2024. Published by The Company of Biologists under the terms of the Creative Commons Attribution License (https://creativecommons.org/licenses/by/4.0/).

The article presented by Akhtar et al. studies the influence of the beginning of the 3Â'UTR part on the expression of the FEM1 gene. To do this, the authors deleted this sequence immediately downstream of the physiological stop codon and then showed an increase in the expression of FEM1. Interestingly, the effect of this deletion is similar to overexpression of FEM1, namely a slowdown in cell growth. This region of the 3'UTR part is very conserved during evolution with the particularity that in humans and chimpanzees, the first stop codon in phase after the physiological stop codon is located 81 nucleotides downstream and for the others species with 51 nucleotides. This conserved sequence destabilizes the synthesized protein and is sufficient to induce accelerated degradation of the protein when added downstream of a physiological stop codon. This sequence plays two roles: facilitating the readthrough of the physiological stop codon and inducing degradation of the protein which contains this sequence. The overall idea of the article is to show that high expression of FEM1 is associated with a better survival rate in cancers.

Comments for the author

This is a very interesting study with conclusions that are supported by the results shown. I think the study is very well conducted and presented in a logical manner. I did not detect a problem with the experiments shown or the need for additional experiments. As far as I am concerned, the manuscript can be accepted as currently presented.

<u>Reviewer 2</u>

Advance summary and potential significance to field

In this study, Akhtar et al. conduct an analysis of the 3'UTR of FEM1B. If the central claim of stop codon readthrough (SCR) regulation of FEM1B could be substantiated, this would constitute a major advance, in cell biology, molecular biology, and oncology.

Comments for the author

Initially, the manuscript appears cohesive, but upon closer examination, several issues emerge. In my assessment, the central claim lacks sufficient support, and there are inconsistencies in the experimental rationale.

The study suggests that a deletion in the 3'UTR stabilizes the protein, in line with the known pro-proliferative functions of FEM1B. However, the subsequent experiments inadequately explore the more plausible explanation that stabilization occurs at the mRNA level (stability). Instead, the authors propose stop codon readthrough (SCR) without providing direct evidence. The evidence for SCR (Figure 4), such as cell staining, reporter assays, and in vitro translation is indirect. Notably, Figure 5C, demonstrating protein stabilization in the presence of MG132, reveals higher molecular bands inconsistent with expected readthrough bands, potentially originating from ubiquitinated forms of FEM1B.
Additionally, the varying molecular weights of constructs in Figures 5B and C suggest experimental discrepancies.

The similarity between the stop codon and mRNA sequence in the UTR of all FEM1B isoforms raises questions about how the stopping ribosome discerns when to support readthrough. The authors propose a hominini-specific hairpin structure lacking empirical evidence and appearing entirely speculative.

It is known that the UGA stop codon is the leakiest of the three stop codons.
The FEM1B stop codon is TAA. How this should support up to 50 or 60% SCR is mysterious. The authors, being experts on SCR, should explain.

Even if we momentarily accept SCR as valid, several incongruities persist. The claim that protein-destabilizing readthrough would affect the non-expanded protein remains unclear. Moreover, there is a lack of evidence for regulation.
Assertions like 'Our results show that the proximal 3'UTR of FEM1B regulates its expression' (l. 417) or 'Thus, it appears that SCR of FEM1B serves as a mechanism evolved to partially relieve the cell

© 2024. Published by The Company of Biologists under the terms of the Creative Commons Attribution License (https://creativecommons.org/licenses/by/4.0/).

cycle from the clutches of FEM1B' (l. 434) or even or even ‘This could partly explain why humans are more susceptible to cancer than other primates‘ (l. 441) lack substantiation. Such claims are unsupported since the only concrete evidence indicates higher expression without the UTR, with no signs of regulation.

Lastly, the connection to cancer would be crucial, but Figure 8 may be misleading. The results in Figure 8A and B are expected due to elevated FEM1B levels, while Figures 8C and D pertain to the expression levels of FEM1B and one of its targets, unrelated to SCR.

In addition to these major concerns, there are several minor points that warrant attention.

* In Figures 2, 3, 5 and 6, biological replicates are normalized in individual pairs and not as a group, creating the impression that the control shows no variance.

* The apparent mol weight of FEM1B differs between various western blots Fig2A: ~72kDa, Fig3A: ~73kDa plus a second band at around 90 kDa, Fig5a: <70kDa, Fig6A = 75k Da

* Some Western Blots (especially in Figure 3) look problematic, eg. too thin actin or H2B bands. Full, unedited blots should be shown. In Figure 3A, the blots should even be redone for detection of distinct bands

* Lines 145-147: Not clear why 38 nucleotides been removed from Δ3’UTR and not the full 81 nucleotide extension?

* Have predicted off-targets of the UTR deletion been analyzed to exclude an influence of any other genes?

* Lines 188-194: the reduction in the expression of H2B and H4 is mentioned without any data of H4 in the associated figures.

* Why does the experimental readthrough efficiency vary that greatly between the in cellulo and in vitro approach? Authors should explain and give absolute RLuc values to exclude variations arising in the normalization.

* Figure 2H: I doubt that the difference in the G2/M phase between pIRES and pIRES-FEM1B is significant with a p-value of 0.004. Raw data are required.

* Figure 4C: The FLuc/RLuc ratios could be normalized to the corresponding 100% readthrough variant (and labelled on the y-axis) since the corresponding percentages appear the text (l. 275).

* Figure 5A-B: In panel A, FEM1Bx is indeed strongly reduced but in in panel B (right side) FEM1Bx expression seems to be stabilized at timepoint 0. Relative density of three individual repetitions may be useful as this has also been done for all other Western blots

* Figures 1B and 6C: The WT HeLa cells show a strongly varying cell proliferation rate in the different experiments (6-fold proliferation in Figure 1 vs. 20-fold proliferation in Figure 6). The experiment in Figure 6C should be repeated with the HeLa Δ3’UTR cells to show the influence of the ISR on cell proliferation in general.

* Figure 8C and D: different labeling of x-axis – unify labeling

* References: Several missing page or article numbers: Chen, Dubiel, Mayr, etc.

First revision

© 2024. Published by The Company of Biologists under the terms of the Creative Commons Attribution License (https://creativecommons.org/licenses/by/4.0/).

Author response to reviewers' comments

Reviewers' comments are in bold and our *responses* are in italics

REVIEWER 1

Advance Summary and Potential Significance to Field:

The article presented by Akhtar et al. studies the influence of the beginning of the 3'UTR part on the expression of the FEM1 gene. To do this, the authors deleted this sequence immediately downstream of the physiological stop codon and then showed an increase in the expression of FEM1. Interestingly, the effect of this deletion is similar to overexpression of FEM1, namely a slowdown in cell growth. This region of the 3'UTR part is very conserved during evolution with the particularity that in humans and chimpanzees, the first stop codon in phase after the physiological stop codon is located 81 nucleotides downstream and for the others species with 51 nucleotides. This conserved sequence destabilizes the synthesized protein and is sufficient to induce accelerated degradation of the protein when added downstream of a physiological stop codon. This sequence plays two roles: facilitating the readthrough of the physiological stop codon and inducing degradation of the protein which contains this sequence. The overall idea of the article is to show that high expression of FEM1 is associated with a better survival rate in cancers.

Reviewer 1 Comments for the Author:

This is a very interesting study with conclusions that are supported by the results shown. I think the study is very well conducted and presented in a logical manner. I did not detect a problem with the experiments shown or the need for additional experiments. As far as I am concerned, the manuscript can be accepted as currently presented.

Response: We thank the Reviewer for recognizing the importance of the study and appreciating our work.

REVIEWER 2

Advance Summary and Potential Significance to Field:

In this study, Akhtar et al. conduct an analysis of the 3'UTR of FEM1B. If the central claim of stop codon readthrough (SCR) regulation of FEM1B could be substantiated, this would constitute a major advance, in cell biology, molecular biology, and oncology.

Response: We thank the Reviewer for acknowledging the significance of our study and for the constructive comments and suggestions. As described below, we have provided multiple evidence for SCR in FEM1B.

Comments for the Author:

Initially, the manuscript appears cohesive, but upon closer examination, several issues emerge. In my assessment, the central claim lacks sufficient support, and there are inconsistencies in the experimental rationale.

Response: As suggested by the Reviewer, we have provided multiple evidence (two types of reporter based assays, mass spectrometry data, ribosome profiling data and detection of the SCR product by western blot) for the central claim (i.e., SCR). We have also addressed the inconsistency issues raised by the Reviewer.

The study suggests that a deletion in the 3'UTR stabilizes the protein, in line with the known pro-proliferative functions of FEM1B. However, the subsequent experiments inadequately explore the more plausible explanation that stabilization occurs at the mRNA level (stability).

Response: We agree that the mRNA stability must be investigated. And, we have explored this possibility using FEM1B Δ3'UTR HeLa cells (Fig 2B) and FEM1B Δ3'UTR MDA-MB-231 cells (Fig S3G). We didn't observe any significant change in the stability of FEM1B mRNA compared to their parental wild-type cells.

Instead, the authors propose stop codon readthrough (SCR) without providing direct evidence. The evidence for SCR (Figure 4), such as cell staining, reporter assays, and in vitro translation is indirect.

Response: We considered SCR as a possibility only after ruling out mRNA stability as described in the response to above question (Fig 2B and S3G). We have provided direct evidence by mass

© 2024. Published by The Company of Biologists under the terms of the Creative Commons Attribution License (https://creativecommons.org/licenses/by/4.0/).

spectrometry and ribosome profiling data analyses (Fig S6 Fig S7). In the revised manuscript, we have provided additional evidence using an antibody that specifically recognizes the SCR product of FEM1B (i.e, FEM1Bx) (Fig S7).

Notably, Figure 5C, demonstrating protein stabilization in the presence of MG132, reveals higher molecular bands inconsistent with expected readthrough bands, potentially originating from ubiquitinated forms of FEM1B.
Response: We regret the confusion here. Figure 5C shows the exogenous expression of tagged FEM1Bx. To achieve this, we have mutated the stop codon of FEM1B such that there will be constitutive expression of FEM1Bx (explained in Results section, lines 277 to 280). This construct does not express FEM1B, it expresses only FEM1Bx. Higher molecular bands the Reviewer is referring to could be ubiquitinated forms of FEM1Bx (not FEM1B).

Additionally, the varying molecular weights of constructs in Figures 5B and C suggest experimental discrepancies.
Response: The apparent discrepancies observed in these Figures are because of the differences in the duration and % of gel used in SDS PAGE. For example, in Fig 5B, the SDS PAGE was performed for longer duration (longer separation of 75 and 50 kDa can be observed) compared to that shown in Fig 5C. These differences do not change the results or their interpretation. We have provided the raw uncropped images of all western blots.

The similarity between the stop codon and mRNA sequence in the UTR of all FEM1B isoforms raises questions about how the stopping ribosome discerns when to support readthrough.
Response: This is an important question. Currently, we are investigating the mechanism of FEM1B readthrough. In general, readthrough is usually driven by transient molecular roadblocks of ribosomes. They can be a cis-acting RNA structure (e.g., pseudoknot or stem-loop) or trans-acting factors binding the mRNA region immediately downstream of the stop codon (e.g., microRNA or proteins). We are currently investigating all these possibilities. In the present manuscript, we have discussed these possibilities (Discussion, last paragraph).

Our preliminary investigations suggest that the ISR of FEM1B can potentially form a stem-loop structure. Mutations that can disrupt such structure reduce the efficiency of SCR indicating that the stem-loop structure may drive (or regulate) this process. Results are shown in the figure below. It requires more experiments to demonstrate the stem-loop structure and its role in SCR. Hence, we have not included in the manuscript.

NOTE: We have removed unpublished data that had been provided for the referees in confidence.

The authors propose a hominini-specific hairpin structure, lacking empirical evidence and appearing entirely speculative.
Response: We regret a misunderstanding here. We have not proposed hominini- specific hairpin structure for FEM1B readthrough.

It is known that the UGA stop codon is the leakiest of the three stop codons. The FEM1B stop codon is TAA. How this should support up to 50 or 60% SCR is mysterious. The authors, being experts on SCR, should explain.
Response: We agree that UGA is the leakiest of the stop codons. To test this in FEM1B context, we mutated the FEM1B stop codon UAA to other two stop codons and measured readthrough efficiency. As expected, and in agreement with the Reviewer, the SCR was highest when UAA was mutated to UGA (Figure S5D).

The high levels of SCR efficiency even across UAA could be due to cis- or trans- acting factors that act at the downstream region of the stop codon. We have discussed this in the manuscript (Discussion, last paragraph).

Even if we momentarily accept SCR as valid, several incongruities persist. The claim that protein-destabilizing readthrough would affect the non-expanded protein remains unclear.
Response: We would like to explain this with an abstract quantitative example. Suppose there are 100 FEM1B mRNA molecules in a cell, and during translation 50% of them exhibit SCR. So, after one round of translation, there will be 50 FEM1B (stable isoform) and 50 FEM1Bx (which will be degraded quickly). So, the net result is 50 FEM1B.

If the SCR is completely stopped due to a deletion (as in case □3'UTR cells described in

© 2024. Published by The Company of Biologists under the terms of the Creative Commons Attribution License (https://creativecommons.org/licenses/by/4.0/).

our manuscript), the 100 FEM1B mRNA molecules will generate 100 FEM1B proteins. So, the net result is 100 FEM1B.

As one can clearly notice, SCR has reduced the generation of FEM1B protein (stable isoform) by 50% in this hypothetical example. Thus, SCR of FEM1B reduces the levels of canonical FEM1B protein by generating an unstable isoform. In a sense SCR renders that particular round of translation unproductive. In the absence of SCR, all mRNAs will generate the stable isoform FEM1B, and its level increases.

Moreover, there is a lack of evidence for regulation.
Response: When the SCR was disrupted by deleting the proximal 3'UTR, the FEM1B protein levels increased (Fig 2A). On the other hand the FEM1B product levels decreased when we made the SCR constitutive (Fig 6A). Together, these results show regulation of FEM1B levels by SCR.

Assertions like 'Our results show that the proximal 3'UTR of FEM1B regulates its expression' (l. 417)
Response: In Fig. 2A and B, we have shown that the absence of the proximal 3'UTR increases the levels of FEM1B protein, without altering its mRNA levels. In Fig 6A, in cells with constitutive SCR (because of stop to sense mutation), we observe decrease in FEM1B protein levels. These observations strongly suggest that there is regulation. However, we do acknowledge the point raised by the Reviewer. And, we have changed the statement to "our results suggest that…."

or 'Thus, it appears that SCR of FEM1B serves as a mechanism evolved to partially relieve the cell cycle from the clutches of FEM1B' (l. 434)
Response: We would like to clarify that it is not an 'assertion', but a mere 'speculation' in Discussion. That's why we write, "it appears…". In this paragraph, we are trying to provide a larger picture for our observations.

or even or even 'This could partly explain why humans are more susceptible to cancer than other primates' (l. 441) lack substantiation. Such claims are unsupported since the only concrete evidence indicates higher expression without the UTR, with no signs of regulation.
Response: We have removed that statement from the Abstract. But retained in the Discussion as a possibility. If the Reviewer insists, we will remove these statements from the manuscript.
As mentioned above, we have provided multiple lines of evidence for SCR.

Lastly, the connection to cancer would be crucial, but Figure 8 may be misleading. The results in Figure 8A and B are expected due to elevated FEM1B levels, while Figures 8C and D pertain to the expression levels of FEM1B and one of its targets, unrelated to SCR.
Response: We agree that the Figure 8 is related to the levels of FEM1B. That is exactly what we claim, "Together, these observations demonstrate a role of FEM1B levels in carcinogenesis" (Last line in Results section). As SCR of FEM1B mRNA regulates FEM1B protein levels, we have included these results. If the Reviewer still feels that the analyses are not suitable here, we will remove them (Fig 8C and D).

In addition to these major concerns, there are several minor points that warrant attention.
In Figures 2, 3, 5 and 6, biological replicates are normalized in individual pairs and not as a group, creating the impression that the control shows no variance.
Response: This is an accepted way of representing densitometry of western blot images. Following are a few examples from the Journal of Cell Science where biological replicates are normalized in individual pairs (similar to what we have done):

1. *Journal of Cell Science (2024) 137, jcs261468. doi:10.1242/jcs.261468 Figure 7D; 8B*
2. *Journal of Cell Science (2024) 137, jcs261476. doi:10.1242/jcs.261476 Figure 1A,D.*
3. *Journal of Cell Science (2024) 137, jcs261047. doi:10.1242/jcs.261047 Figure 2B, E*

The variance shown in the graphs represents variation in the fold change. That should not be confused as variation (or lack of it) in the western blot densities.

The apparent mol weight of FEM1B differs between various western blots Fig2A:

© 2024. Published by The Company of Biologists under the terms of the Creative Commons Attribution License (https://creativecommons.org/licenses/by/4.0/).

~72kDa, Fig3A: ~73kDa plus a second band at around 90 kDa, Fig5a: <70kDa, Fig6A = 75k Da
Response: As mentioned above, the apparent discrepancies observed in those Figures are because of the differences in the duration and % of gel used in SDS PAGE. For example, in Fig 5B, the SDS PAGE was performed for longer duration (longer separation of 75 and 50 kDa can be observed) compared to that shown in Fig 5C. These differences do not change the results or their interpretation. We have provided raw uncropped images of all western blots.

Some Western Blots (especially in Figure 3) look problematic, eg. too thin actin or H2B bands. Full, unedited blots should be shown. In Figure 3A, the blots should even be redone for detection of distinct bands
Response: We have provided uncropped raw images of all western blots in Supplementary Information. We have replaced the actin blot with the one with denser and distinct bands in 3A (higher exposure).

Lines 145-147: Not clear why 38 nucleotides been removed from Δ3'UTR and not the full 81 nucleotide extension?
Response: We designed two sgRNAs (sequences provided in Methods) with minimal off-target score to target the proximal 3'UTR of FEM1B. This is because our initial objective was to understand the importance of this conserved region. When transfected with Cas9, these two sgRNAs resulted in 38-nucleotide deletion in HeLa cells (Fig 1A).

Have predicted off-targets of the UTR deletion been analyzed to exclude an influence of any other genes?
Response: Yes, we have. The sgRNAs were selected based on their high target score and low off-target score compared to others using Benchling software. Poor off-target tendency was confirmed by BLAST also. We have provided this information in Methods under "Deletion of the proximal part of the 3'UTR of FEM1B"

Lines 188-194: the reduction in the expression of H2B and H4 is mentioned without any data of H4 in the associated figures.
Response: The H4 data is provided in supplementary figures (Fig S4). Somehow, the Reviewer has missed this.

Why does the experimental readthrough efficiency vary that greatly between the in cellulo and in vitro approach? Authors should explain and give absolute RLuc values to exclude variations arising in the normalization.
Response: This is a very good point. At this point, we don't know the exact reason. We speculate that there could be some trans-acting factors (proteins or microRNAs) that regulate the process of SCR inside the cells, which are absent in vitro. We have included this explanation in the revised manuscript (Lines 267-268). More investigations are required to completely understand this.

As suggested by the Reviewer, we have provided the raw values of luciferase readings in Supplementary Information file.

Figure 2H: I doubt that the difference in the G2/M phase between pIRES and pIRES-FEM1B is significant with a p-value of 0.004. Raw data are required.
Response: We have rechecked the values and the statistics. The P value is 0.0065.
Actual values are given below:

pIRES	pIRES FEM1B
43.17	50.42
42.66	52.34
44.70	45.89
47.11	52.30
46.61	50.23

Figure 4C: The FLuc/RLuc ratios could be normalized to the corresponding 100% readthrough variant (and labelled on the y-axis) since the corresponding percentages appear the text (l.

© 2024. Published by The Company of Biologists under the terms of the Creative Commons Attribution License (https://creativecommons.org/licenses/by/4.0/).

275).
Response: As per this suggestion, we have modified the graphs to show % of readthrough in the Y axis (Fig 4C).

Figure 5A-B: In panel A, FEM1Bx is indeed strongly reduced but in in panel B (right side) FEM1Bx expression seems to be stabilized at timepoint 0.
Response: The densities of FEM1Bx bands in 5A and 5B cannot be compared as the exposure time was different. To be specific, the exposure time in 5B was much longer than that in 5A. That's why we see a denser band.

Relative density of three individual repetitions may be useful as this has also been done for all other Western blots
Response: As per this suggestion, we now have included the densitometry data (Fig 5A).

Figures 1B and 6C: The WT HeLa cells show a strongly varying cell proliferation rate in the different experiments (6-fold proliferation in Figure 1 vs. 20-fold proliferation in Figure 6).
Response: The calculated fold proliferation is highly sensitive to initial number of cells. A small change in the initial numbers result in large change in the fold proliferation. This is the reason for variation in the fold proliferation.

The experiment in Figure 6C should be repeated with the HeLa Δ3'UTR cells to show the influence of the ISR on cell proliferation in general.
Response: As per this suggestion we repeated the experiment with Δ3'UTR cells and the results are shown in the updated Fig 6C.
Figure 8C and D: different labeling of x-axis - unify labelling
Response: We have now made the X-axis label uniform (i.e., Years)

References: Several missing page or article numbers: Chen, Dubiel, Mayr, etc. *Response: We thank the Reviewer for identifying these mistakes. We have corrected them.*

<u>Second decision letter</u>

MS ID#: JOCES/2023/261921

MS TITLE: Hominini-specific regulation of cell cycle by stop codon readthrough of *FEM1B*

AUTHORS: Md Noor Akhtar, Anumeha Singh, Lekha Manjunath, Dhruba Dey, Sangeetha Devi Kumar, Kirtana Vasu, Arpan Das, and Sandeep Eswarappa

We have now reached a decision on the above manuscript.

To see the reviewers' reports and a copy of this decision letter, please go to: https://submit-jcs.biologists.org and click on the 'Manuscripts with Decisions' queue in the Author Area. (Corresponding author only has access to reviews.)

As you will see, one reviewer raise a number of substantial criticisms that prevent me from accepting the paper at this stage. In particular this reviewer considers that using anti-FAM1B antibodies together with anti-FAM1Bx antibodies on Western blots of wild-type cells is essential to provide an estimation of relative quantities of forms with and without readthrough. This reviewer also questions whether this phenomenon is solely occurring in HepG2 cells. If you think that you can deal satisfactorily with the criticisms on revision, I would be pleased to see a revised manuscript. We would then return it to this reviewer.

Please ensure that you clearly highlight all changes made in the revised manuscript. Please avoid using 'Tracked changes' in Word files as these are lost in PDF conversion.

© 2024. Published by The Company of Biologists under the terms of the Creative Commons Attribution License (https://creativecommons.org/licenses/by/4.0/).

I should be grateful if you would also provide a point-by-point response detailing how you have dealt with the points raised by the reviewers in the 'Response to Reviewers' box. Please attend to all of the reviewers' comments. If you do not agree with any of their criticisms or suggestions please explain clearly why this is so.

Reviewer 1

Advance summary and potential significance to field

The article presented by Akhtar et al. studies the influence of the beginning of the 3Â'UTR part on the expression of the FEM1 gene. To do this, the authors deleted this sequence immediately downstream of the physiological stop codon and then showed an increase in the expression of FEM1. Interestingly, the effect of this deletion is similar to overexpression of FEM1, namely a slowdown in cell growth. This region of the 3'UTR part is very conserved during evolution with the particularity that in humans and chimpanzees, the first stop codon in phase after the physiological stop codon is located 81 nucleotides downstream and for the others species with 51 nucleotides. This conserved sequence destabilizes the synthesized protein and is sufficient to induce accelerated degradation of the protein when added downstream of a physiological stop codon. This sequence plays two roles: facilitating the readthrough of the physiological stop codon and inducing degradation of the protein which contains this sequence. The overall idea of the article is to show that high expression of FEM1 is associated with a better survival rate in cancers.

Comments for the author

With the suggestions of the second reviewer, the manuscript seems even more acceptable for publication. For me the results are convincing and support the conclusions.

Reviewer 2

Advance summary and potential significance to field

See below

Comments for the author

The authors have made significant efforts to address the reviewer's requests.
However, it is regrettable to note that a substantial gap persists within the data, and there is insufficient evidence to support the assertion of translational readthrough in this context.

For instance, in Figure S7, it appears that readthrough addition is only observable in HepG2 cells, with limited or absent expression in the majority of other cell types utilized in this study. Furthermore, some cell types were not subjected to testing at all. Employing anti-FAM1B antibodies in conjunction with anti-FAM1Bx antibodies on Western blots of wild-type cells could facilitate the estimation of relative quantities of forms with and without readthrough. Yet the authors have not presented any blots utilizing these antibodies on the same sample. Conditions designed to stimulate readthrough should theoretically result in a higher signal of the x-form, among other expectations.

Regrettably, I cannot endorse the manuscript for publication, as there is a significant likelihood that the central claim of the manuscript is fundamentally flawed. Publishing under such circumstances would be detrimental both to the authors and to the reputation of JCS.

I am aware that this assessment may appear severe, potentially leading to disappointment for the authors if taken seriously by the editors. To support this decision, I have reached out to experts beyond my own expertise, e.g. in Ribo-Seq. Importantly, I have done so without compromising the authors' anonymity or divulging any detail from the manuscript. These colleagues have corroborated the absence of evidence supporting readthrough in FAM1B.

© 2024. Published by The Company of Biologists under the terms of the Creative Commons Attribution License (https://creativecommons.org/licenses/by/4.0/).

Second revision

Author response to reviewers' comments

Reviewers' comments are in bold and our *responses* are in italics

REVIEWER 1

Advance Summary and Potential Significance to Field:

The article presented by Akhtar et al. studies the influence of the beginning of the 3'UTR part on the expression of the FEM1 gene. To do this, the authors deleted this sequence immediately downstream of the physiological stop codon and then showed an increase in the expression of FEM1. Interestingly, the effect of this deletion is similar to overexpression of FEM1, namely a slowdown in cell growth. This region of the 3'UTR part is very conserved during evolution with the particularity that in humans and chimpanzees, the first stop codon in phase after the physiological stop codon is located 81 nucleotides downstream and for the others species with 51 nucleotides. This conserved sequence destabilizes the synthesized protein and is sufficient to induce accelerated degradation of the protein when added downstream of a physiological stop codon. This sequence plays two roles: facilitating the readthrough of the physiological stop codon and inducing degradation of the protein which contains this sequence. The overall idea of the article is to show that high expression of FEM1 is associated with a better survival rate in cancers.

Comments for the Author:

With the suggestions of the second reviewer, the manuscript seems even more acceptable for publication. For me the results are convincing and support the conclusions.

Response: We thank the Reviewer for the positive comments

REVIEWER 2

The authors have made significant efforts to address the reviewer's requests. However, it is regrettable to note that a substantial gap persists within the data, and there is insufficient evidence to support the assertion of translational readthrough in this context.

Response: We would like to reiterate that we have provided multiple lines of evidence for the SCR of FEM1B by using all the key techniques and tools currently used in this field as listed below:

1. *Dual luciferase based readthrough assay, which is considered as a gold standard for readthrough and other recoding processes (Fig. 4C).*
2. *Single luciferase-based readthrough assay (Fig. S5).*
3. *Fluorescence-based readthrough assay (Fig. 4B).*
4. *Detection of readthrough protein from an exogenous construct (Fig S7B).*
5. *Detection of endogenous readthrough protein, FEM1Bx, using a specific antibody (Fig S7C, D and E).*
6. *Detection of peptides encoded by the region after the stop codon (ISR) by analysing mass-spectrometry data (Fig S6A and S7F)*
7. *Detection of ribosomal footprints after the stop codon of FEM1B both in global ribosome profile and tissue/cell-specific profile (Fig S6B, C and D)*

Evidence from experiments 1 to 4 demonstrate SCR in exogenous constructs, and evidence from experiments and analyses mentioned in 5 to 7 show SCR of endogenous FEM1B. Among these, the data for mass-spectrometry analyses and ribosome footprint analyses were obtained from publicly available databases deposited by other research groups.

Therefore, we would like to state that we have provided sufficient evidence to support our claim that FEM1B exhibits stop codon readthrough.

For instance, in Figure S7, it appears that readthrough addition is only observable in HepG2 cells, with limited or absent expression in the majority of other cell types utilized in this study.

Response: Yes, the Reviewer's interpretation is correct. FEM1Bx was detectable in HepG2 cells,

© 2024. Published by The Company of Biologists under the terms of the Creative Commons Attribution License (https://creativecommons.org/licenses/by/4.0/).

not in other cell types that were tested. This was also the case in mass spectrometry data analysis (Fig S7F). Though we analysed mass-spectrometry data from multiple cell lines, the FEM1Bx-specific peptide was detected only in HepG2 cells. Undetectable levels of FEM1Bx in most cell lines tested can be explained by its poor stability, which we have demonstrated (Fig 5). Its detection in HepG2 cells suggest that the readthrough isoform escapes proteasomal degradation in HepG2 cells. The mechanism requires more investigations.

Furthermore, some cell types were not subjected to testing at all.
Response: We have tested the expression of FEM1Bx in HEK293, HeLa, HepG2, PANC-1, MDA-MB-231 and MiaPaCa-2 cells. These cells are derived from different human tissues (kidney, cervix, liver, pancreas and breast). It is not clear to us which cell types the Reviewer refers to.

Employing anti-FAM1B antibodies in conjunction with anti-FAM1Bx antibodies on Western blots of wild-type cells could facilitate the estimation of relative quantities of forms with and without readthrough. Yet, the authors have not presented any blots utilizing these antibodies on the same sample.
Response: We have now included the data (FEM1B expression in <u>same samples</u>) in the revised manuscript (Fig S7C). This was not included in our manuscript as our intention was just to detect the expression of the readthrough product, and we thought the expression of FEM1B (which expectedly varies across the cell types) may not give any insight into the readthrough process.

Conditions designed to stimulate readthrough should theoretically result in a higher signal of the x-form, among other expectations.
Response: We have tested in conditions that abrogate readthrough (which serves this purpose), i.e., in □3′UTR MDA-MB-231 cells, where the mRNA region responsible for readthrough has been deleted. These mutant cells don't show the expression of FEM1Bx, whereas their parental wild-type cells show the expression (Fig S7E). Furthermore, in HepG2 cells transfected with shRNAs targeting FEM1B, we observed much reduced expression of FEM1Bx (Fig S7D). At present we don't know the conditions that stimulate readthrough of FEM1B.

Regrettably, I cannot endorse the manuscript for publication, as there is a significant likelihood that the central claim of the manuscript is fundamentally flawed. Publishing under such circumstances would be detrimental both to the authors and to the reputation of JCS.
Response: We appreciate the concern, but we respectfully and strongly disagree with the opinion of the Reviewer. As described above, our central claim is based on multiple lines of evidence gathered from 7 different assays and analyses used in the field currently.
I am aware that this assessment may appear severe, potentially leading to disappointment for the authors if taken seriously by the editors. To support this decision, I have reached out to experts beyond my own expertise, e.g. in Ribo- Seq. Importantly, I have done so without compromising the authors' anonymity or divulging any detail from the manuscript.These colleagues have corroborated the absence of evidence supporting readthrough in FAM1B.
Response: We really appreciate the efforts of the Reviewer. However, the Reviewer's colleagues' opinion without any data or details of the analyses is not helpful. We need more information to respond to this comment – the type of cells/tissues in which the analysis was done, the method used, the actual profile of ribosome footprints on the FEM1B mRNA, etc.

In the revised manuscript, we have provided more evidence. In addition to global profile of ribosome footprints on FEM1B mRNA, we have included profiles from human testis tissue (shows very high expression of FEM1B according to Protein Atlas) and HeLa cells. The profiles show footprints after the stop codon strengthening our claim on stop codon readthrough of FEM1B (Fig S6C and D).

<u>Third decision letter</u>

MS ID#: JOCES/2023/261921

MS TITLE: Hominini-specific regulation of cell cycle by stop codon readthrough of *FEM1B*

© 2024. Published by The Company of Biologists under the terms of the Creative Commons Attribution License (https://creativecommons.org/licenses/by/4.0/).

AUTHORS: Md Noor Akhtar, Anumeha Singh, Lekha Manjunath, Dhruba Dey, Sangeetha Devi Kumar, Kirtana Vasu, Arpan Das, and Sandeep Eswarappa

I am happy to tell you that your manuscript has been accepted for publication in Journal of Cell Science, pending standard publication integrity checks.

© 2024. Published by The Company of Biologists under the terms of the Creative Commons Attribution License (https://creativecommons.org/licenses/by/4.0/).
